# Leucine-rich *α*-2-glycoprotein 1 initiates the onset of diabetic retinopathy in mice

**DOI:** 10.1126/scitranslmed.adn6047

**Published:** 2025-10-22

**Authors:** Giulia De Rossi, Ao-wang Qiu, Maxime Berg, Thomas Burgoyne, Andrea Martello, Marlene E. Da Vitoria Lobo, Matteo Rizzi, Sophie Mueller, Jack Blackburn, Yuxuan Meng, Simon Walker-Samuel, Rebecca Shipley, Colin J. Chu, Sobha Sivaprasad, John Greenwood, Stephen E. Moss

**Affiliations:** 1 Institute of Ophthalmology, University College London; EC1V 9EL London (UK); 2 Department of Mechanical Engineering, University College London; WC1E 7JE London (UK); 3 Centre for Computational Medicine, University College London; WC1E 6JF London (UK); 4 NIHR Moorfields Biomedical Research Centre, Moorfields Eye Hospital; EC1V 2PD London (UK); 5 Institut de Pharmacologie et de Biologie Structurale (IPBS), Université de Toulouse, CNRS, Université Toulouse III - Paul Sabatier (UT3), 31077 Toulouse, France; 6 Department of Ophthalmology, The First Affiliated Hospital of Nanjing Medical University; Nanjing 210029 China

## Abstract

Diabetic retinopathy (DR) is a common complication of diabetes mellitus and a leading cause of visual impairment and blindness in the working-age population. The early stage of the disease is characterized by retinal capillary dysfunction, but the mechanisms whereby hyperglycemia disturbs capillary homeostasis at this initiating stage are poorly understood, posing a barrier to the development of effective early treatments. We employed two mouse models of type I diabetes that replicate early features of human retinal vascular pathology. In both the streptozotocin (STZ) model, where hypoinsulinemia is chemically induced, and in the Ins2Akita model, which develops it spontaneously due to a mutation in the insulin gene, we observed early induction of the secreted glycoprotein gene leucine-rich *α*-2-glycoprotein 1 (*Lrg1*). Using the Ins2Akita mice, we showed that *Lrg1* induction preceded that of vascular endothelial growth factor A (*Vegfa)*. LRG1 initiated retinal microvascular dysfunction by modifying transforming growth factor β (TGFβ) signaling in pericytes, driving transdifferentiation to a more contractile fibrotic phenotype, resulting in narrower capillaries and thickened basement membrane. Using computational modelling, we showed that these early vascular changes impaired retinal blood flow and oxygen delivery, consistent with a defect in visual transduction observed in both models. This early retinal phenotype could be rescued by *Lrg1* knockout or by treatment with an LRG1 function-blocking antibody in both the STZ and Ins2Akita mice. These results demonstrate that LRG1 is a driver of vascular dysfunction that contributes to the onset of DR and presents itself as a potential pre-emptive therapeutic target.

## Introduction

Diabetic retinopathy (DR) is a prevalent complication of diabetes and one of the main causes of blindness and visual impairment in the working-age population. Characterized by progressive damage to retinal blood vessels accompanied by neurodegeneration, the pathogenesis of DR has been linked to several essential biochemical pathways that are modified by sustained hyperglycemia including hexosamine biosynthesis, protein kinase A, advanced-glycation end products, renin-angiotensin and polyol (1, 2). These independent but interconnected pathways contribute to retinal inflammation and metabolic stress, and vascular cells are among the first to be affected. Clinically, microvascular dysfunction in the early stage of DR manifests with microaneurysms, venous beading, hemorrhages, and spot leakage (3, 4). Concomitantly, early microvascular changes can also cause breakdown of the blood-retinal barrier leading to diabetic macular edema (DME) and visual impairment. In time, the vessel damage results in capillary dropout causing hypoxia and triggering uncontrolled and fragile neovascularization, defining conversion of the disease to the sight-threatening proliferative stage (PDR). Current therapeutic interventions are primarily aimed at restoring vascular stability by blocking vascular endothelial growth factor (VEGF), a pro-permeability and pro-angiogenic cytokine that is upregulated in eyes with increasing severity of DR (5). Although anti-VEGF drugs elicit a good response in around half of patients with DME, their efficacy is often short-lived (4) and prolonged use may be accompanied by unwanted side-effects (6, 7). Moreover, the failure of VEGF blockade to reduce edema in all patients indicates the presence of other disrupting factors yet to be identified. In PDR, anti-VEGF strategies can elicit regression of pathological neo-vessels although this does not address the need for re-establishment of a patent, functioning vascular bed. These observations highlight the need for targeting microvascular changes early. Through the application of optical coherence tomography angiography (OCTA), visualization of the retinal vascular plexi (superficial, intermediate, and deep) has revealed that the superficial and deep vascular density decreases before the onset of DR (8). Moreover, impaired visual function and retinal neuronal degenerative changes may arise before the onset of DR in people with diabetes (9). However, the earliest pathophysiological events that lead to capillary dysfunction in diabetes, which trigger ischemia and the subsequent breakdown of the blood-retinal barrier and neovascularization, remain poorly understood. What is needed, therefore, is greater insight into the causes of the early subtle initiating events, as this will allow the development of interventions that may slow or prevent the disease progressing to later visually damaging stages.

Leucine-rich α-2-glycoprotein 1 (LRG1) is a secreted glycoprotein expressed constitutively by hepatocytes and neutrophils, with little expression by other cell types under non-pathological conditions (10). One of its main modes of action is in modulating transforming growth factor β (TGFβ) signaling, where in disease it plays pivotal roles in both neovascularization (11, 12) and fibrosis (13). It is also involved in immune responses and has been described as a potential acute-phase protein since its hepatic expression is enhanced by systemic inflammation (14). Cross-sectional studies reported LRG1 protein to be increased in plasma (15, 16) and urine (17) of people with diabetes, and proteomic analyses of vitreous humor from patients with DR have revealed that LRG1 is up-regulated (11, 18–21). Whereas pre-clinical studies using oxygen-induced retinopathy (OIR) as a model of retinal neovascularization suggest involvement of LRG1 in the late neovascular stage of DR (11), it is not known whether it could also play a role in the early stage of the disease. Here, we investigated whether LRG1 contributes to early microvascular dysfunction in DR and, if so, whether its inhibition may provide therapeutic benefit.

## Results

### LRG1 is upregulated systemically and locally in the retina during hyperglycemia

**Fig. 1:**
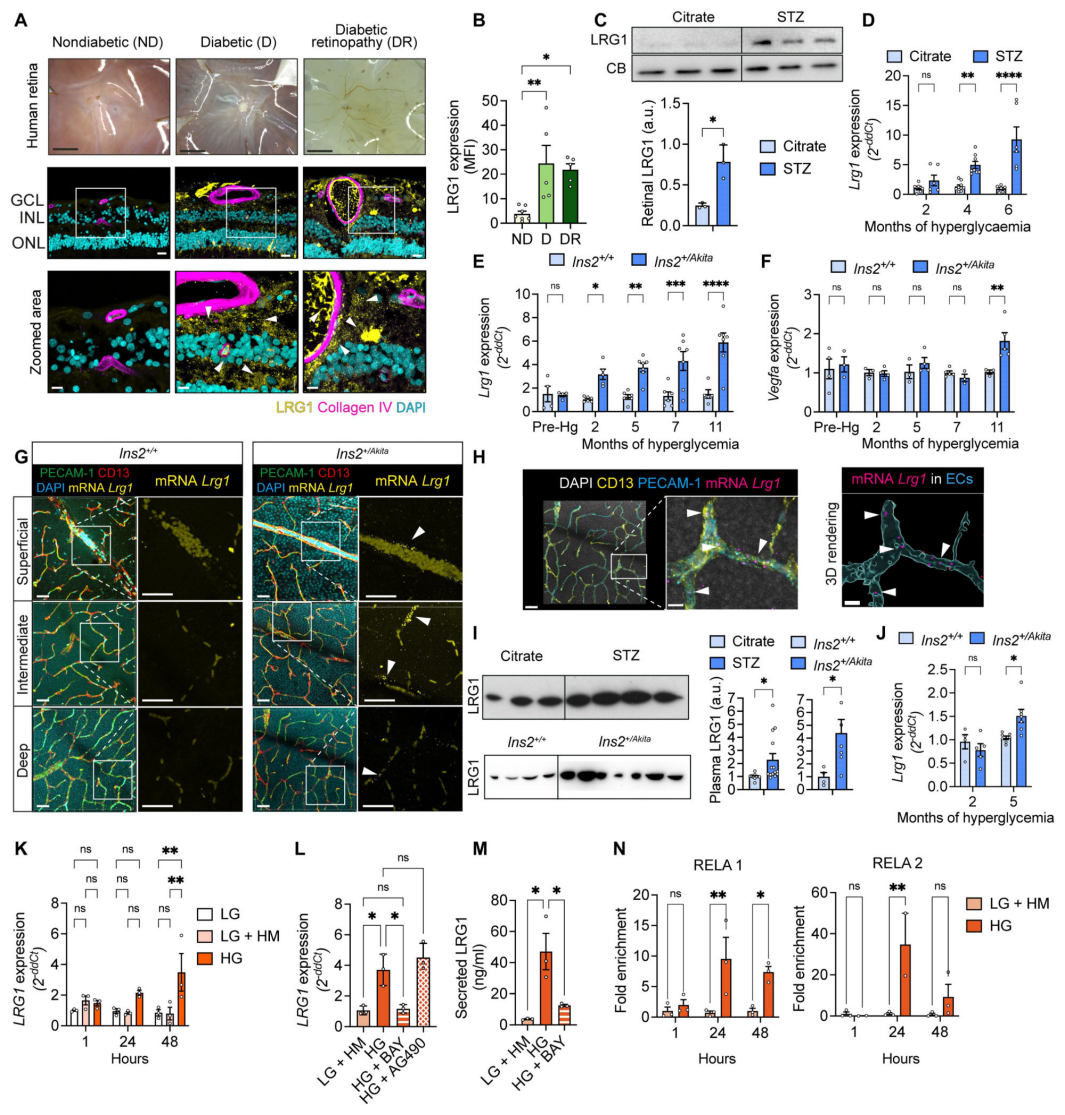
LRG1 expression following hyperglycemia. (**A**) Photographs of human retinas from three representative donors with no diabetes (ND), diabetes (D) or diabetic retinopathy (DR) at dissection (Scale bar, 5 mm) and their respective retinal cross-sections immunostained for LRG1 (yellow), blood vessels (collagen IV, magenta) and nuclei (DAPI, cyan). Arrowheads indicate LRG1 protein in ONL (outer nuclear layer) and GCL (ganglion cell layer). Scale bar, 10 μm. The lower magnification images shown here are reused in fig. S1C to allow comparison with the other donors. (**B**) LRG1 signal quantified as mean fluorescence intensity (MFI), each data point representing one donor. n=7 (ND), 5 (D) and 5 (DR). (**C**) LRG1 expression in retinal lysates from control (citrate) and hyperglycemic (STZ) mice following 6 months of hyperglycemia. n=3 mice per group. Arbitrary units (a.u.) represent the relative expression of LRG1 normalized to the loading control, Cyclophilin B (CB). Unpaired two-tailed t test. (**D**) *Lrg1* expression in retina/RPE from control (Citrate) and diabetic (STZ) mice at the time points indicated. n=6-9 mice per group. 2-way Anova; Šidák’s test for multiple comparisons. (**E**) *Lrg1* and (**F**) *Vegfa* expression in retinas from control (*Ins2^+/+^*) and diabetic (*Ins2^+/Akita^*) mice at different time points following hyperglycemia or one-month prior to onset (Pre-Hg). n=4-7 mice per group. 2-way Anova; Šidák’s test for multiple comparisons. (**G**) RNA in situ hybridization of *Lrg1* mRNA (yellow) and immunostaining of the retinal vasculature of 4-month hyperglycemic Ins2Akita mice and controls. ECs (PECAM-1, green), pericytes (CD13, red) and nuclei (DAPI, cyan). White arrowheads indicate the presence of *Lrg1*^*+*^ puncti. Scale bar, 70 μm. (**H**) Higher magnification of the deep plexus of *Ins2^+/Akita^* mice shown in (G), to highlight the presence of *Lrg1*^+^ puncti within the vasculature. 3D surface rendering to show the inclusion of *Lrg1* transcript within the EC layer. Scale bar, 70 μm. (**I**) Plasma LRG1 in control and diabetic mice from both models after 5 months of hyperglycemia. n=4-7 independent experiments. Arbitrary units (a.u.) represent the relative expression of LRG1 normalized to the control non-diabetic samples. Unpaired two-tailed t test. (**J**) *Lrg1* expression in livers from control (*Ins2^+/+^*) and diabetic (*Ins2^+/Akita^*) mice after 2 and 5 months of hyperglycemia. n=4-7 mice per group. 2-way Anova; Šidák’s test for multiple comparisons. (**K**) *LRG1* expression in HRECs treated for the times indicated with low glucose (5.5 mM glucose, LG), osmolality control (5.5 mM glucose plus mannitol up to 25 mM, LG + HM) or high glucose (25 mM, HG). n=3 independent experiments. 1-way Anova; Tukey’s test for multiple comparisons. (**L**) *LRG1* expression in HRECs treated for 48 hours with low glucose (LG + HM), or high glucose (HG) alone or with 1 μM NF-κB inhibitor BAY 11-7082 or 50 μM STAT3 inhibitor AG490. n=3 independent experiments. 1-way Anova; Šidák’s test for multiple comparisons. (**M**) Secreted LRG1 measured by ELISA in conditioned media from HRECs treated for 48 hours with low glucose (LG + HM) or HG, alone or with 1 μM NF-κB inhibitor BAY 11-7082. n=3 independent experiments. 1-way Anova; Šidák’s test for multiple comparisons. (**N**) ChIP-qPCR analysis of association between NF-κB/p65 and its binding sites (RELA 1 and RELA 2) on the human *LRG1* promoter in HRECs treated for the hours indicated with low (LG + HM) or high glucose (HG). n=3. 2-way Anova; Šidák’s test for multiple comparisons. All bars represent mean and standard error of the mean. P*<0.05; P**<0.01; P***<0.001; P****<0.0001.

Previous cross-sectional studies have documented elevated LRG1 concentration in plasma and vitreous humor of individuals with diabetes (11, 15–21), yet there is limited evidence of LRG1 expression within the retina itself. To address this gap, we analyzed LRG1 expression in post-mortem retinal tissues from donors with or without diabetes (type 1 or 2), including cases diagnosed with DR (table S1). Despite the presence of confounding factors and the heterogeneity in sex and age in the cohorts (table S1), we found that LRG1 was consistently elevated in the retinas from donors with diabetes, regardless of the presence of overt diagnosed retinopathy, compared to those without diabetes (Fig. 1 A and B, fig. S1 A to C, table S1). LRG1 exhibited a punctate and heterogeneous distribution across the tissue, consistent with its expression as a secreted protein. Discrete foci of intense LRG1 immunoreactivity were sporadically detected in diseased tissues. These regions co-localized with plasma markers, suggesting the possibility that a proportion of LRG1 may originate from localized vascular leakage (fig. S1D). To determine whether the diabetic retina contributes to the ectopic expression of *Lrg1*, and to assess whether this is a direct consequence of hyperglycemia, we employed the streptozotocin (STZ) and the Ins2Akita mouse models. Following 6 months of hyperglycemia, we found LRG1 protein increased in the retinas of diabetic mice compared to controls (Fig. 1C). In both models, we observed a time-dependent increase in *Lrg1* transcript in the retinas, evident after 2 and 4 months of hyperglycemia, in the STZ and Ins2Akita mice respectively (Fig. 1, D and E). In Ins2Akita mice, *Lrg1* expression preceded that of *Vegfa* by many months (Fig. 1F), as well as that of hypoxia-inducible factor 1α (*Hif1α*) and *Tgfβ* (fig. S2, A and B), known players in DR progression (22, 23), suggesting that LRG1 might be an early primer of disease. To determine the cellular source of LRG1 in the retina, we combined immunofluorescence with RNA in situ hybridization and examined the three retinal vascular plexi (superficial, intermediate, and deep) guided by the vascular markers platelet/endothelial cell adhesion molecule-1 (PECAM-1) for endothelial cells (ECs) and CD13 for pericytes. Under control conditions (*Ins2^+/+^*), there was negligible expression of *Lrg1*, whereas *Lrg1* expression was evident in the retinal vasculature of hyperglycemic mice (*Ins2^+/Akita^*) (Fig. 1G), mostly associated with ECs (Fig. 1H). To determine whether the increase in *Lrg1* in hyperglycemia is restricted to the retina or is also systemic, plasma LRG1 from STZ and Ins2Akita mice was measured at 5 months. We found LRG1 in the plasma of control mice and elevation after 5 months of hyperglycemia (Fig. 1I), with possible contribution from a small increase in liver *Lrg1* expression (Fig. 1J). In accordance with a vascular origin for mouse retinal LRG1, as indicated by RNA in situ hybridisation (Fig. 1H), cultured human retinal ECs (HRECs) exhibited enhanced *LRG1* expression after 48 hours conditioning with high glucose (Fig. 1K). To identify the pathway mediating this response, we focused on signal transducer and activator of transcription 3 (STAT3) and nuclear factor *κ*-light-chain-enhancer of activated B (NF-κB), since the promoter for the human *LRG1* gene contains binding sites for both transcription factors (fig. S3A) and a link between these two pathways and *LRG1* expression has already been demonstrated (24, 25). Critically, both inflammatory pathways are active in human diabetic retinas (26). By using NF-κB (BAY11-7082) and interleukin 6 (IL-6)/STAT3 (AG490) inhibitors, we showed that high glucose increased *LRG1* expression in HRECs through the NF-κB pathway (Fig. 1, L and M) and that this transcription factor directly bound to the *LRG1* promoter (Fig. 1N). A direct effect of high glucose on NF-κB activation has been well documented (27–31). Consistent with this, we demonstrated that vascular cell adhesion protein 1 (VCAM-1), a known NF-κB target gene, is also upregulated in HRECs exposed to high glucose (fig. S3B) and in the retinas of hyperglycemic mice (fig. S3C), confirming NF-κB activation under our experimental conditions. Collectively, these findings show that LRG1 expression is upregulated in the retina during diabetes, with hyperglycemia driving this effect in inflamed retinal ECs through NF-κB activation.

### LRG1 promotes onset of vascular pathology in murine models of DR

**Fig. 2:**
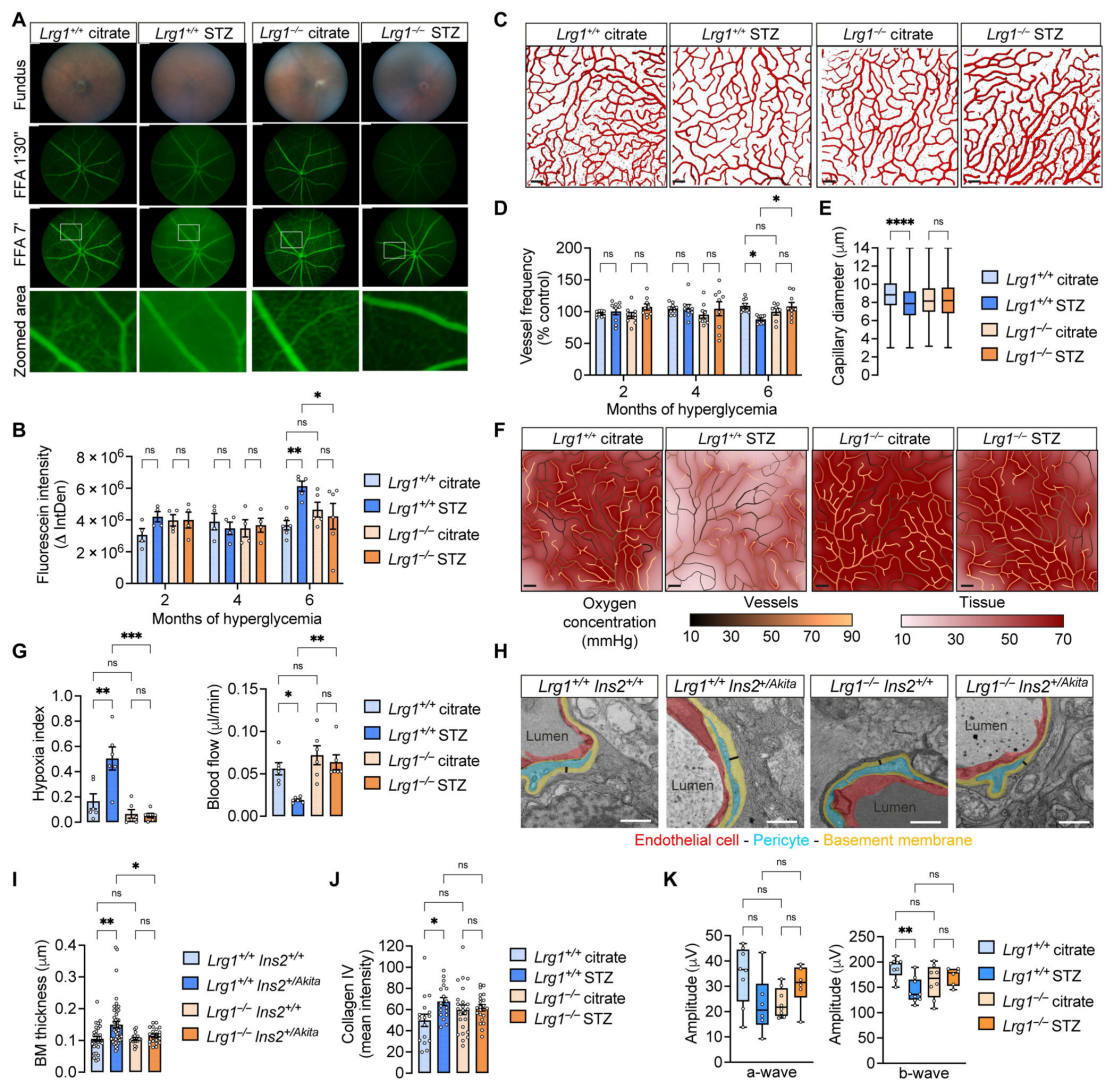
LRG1 promotes vascular dysfunction in the hyperglycemic retina. (**A**) Representative fundus images from STZ diabetic mice at 6 months post-hyperglycaemia and age-matched controls. (**B**) FFA angiograms of the same mice after 90 sec and 7 min following subcutaneous fluorescein injection. Leakage expressed as delta integrated density (ΔIntDen) of fluorescence between the two time points. n=4-6 mice per group. 2-way Anova; Tukey’s test for multiple comparisons. (**C**) Confocal images of the retinal deep-plexus of STZ diabetic mice at 6 months post-hyperglycaemia and age-matched controls. PECAM-1^+^ ECs (red). Scale bar, 50 μm. (**D**) Vessel density expressed as the number of vessels crossing an arbitrary line, normalized to control (2 months, *Lrg1^+/+^* citrate). n=7-9 mice per group. 2-way Anova; Tukey’s test for multiple comparisons. (**E**) Capillary diameter from images of segmented vasculature. (**F**) Image-based computational analysis of oxygen delivery. Representative images show simulated oxygen concentration in the tissue and within the vasculature. (**G**) Quantification of the oxygen extraction coefficient and blood flow. n=6 mice per group. 1-way Anova; Tukey’s test for multiple comparisons. (**H**) TEM images of retinas from control (*Ins2^+/+^*) and diabetic (*Ins2^+/Akita^*) mice on a *Lrg1^+/+^* or *Lrg1^-/-^* background after 6 months of hyperglycaemia. EC (red), pericyte (cyan) and basement membrane (yellow). Scale bar, 1 μm. (**I**) Basement membrane thickness of deep-plexus capillaries measured manually by drawing a perpendicular line above the pericyte (black bar). n=3 mice per group, images≥17 per mouse. 1-way Anova; Tukey’s test for multiple comparisons. (**J**) Quantification of Collagen IV mean expression across the retinal vasculature of STZ mice following 6 months of hyperglycemia. (**K**) ERG response after short dark adaptation measured in the 4 groups after 6 months of hyperglycemia. n=6-9 mice per group. 1-way Anova; Tukey’s test for multiple comparisons. All data in bar charts represent mean and standard error of the mean. Whiskers in box plots extend to min and max values, box extends from the 25th to 75th percentiles and the line in the middle of the box is plotted at the median. P*<0.05; P**<0.01; P***<0.001; P****<0.0001.

We next sought to determine if LRG1 is causally linked to early DR pathogenesis. Both STZ and Ins2Akita mice develop a retinal vascular phenotype similar to that reported early in human DR (32, 33) and are therefore ideal to test this hypothesis. We first verified that *Lrg1^-/-^* mice display blood glucose concentrations and body weights comparable to those of wild-type littermates in both models (fig. S4, A and B) before assessing readouts of vascular dysfunction after 2-6 months of hyperglycemia. Retinal hyperpermeability, evaluated by fundus fluorescein angiography, showed increased dye leakage and loss of intracapillary fluorescein only in wild-type *Lrg1^+/+^* STZ mice after 6 months of hyperglycemia, whereas retinal permeability in *Lrg1^-/-^* mice was unchanged at all 3 time points studied (Fig. 2, A and B). Given this association between LRG1 and early loss of vascular integrity, we investigated its impact on the vascular architecture. Confocal analysis of the deep vascular plexus revealed a decrease in vascular density and volume in wild-type animals after 6 months of hyperglycemia, and no changes were observed in *Lrg1^-/-^* retinas (Fig. 2, C and D, fig. S4C). Moreover, measurement of retinal capillary diameter revealed subtle but significant (P<0.0001) capillary vasoconstriction in hyperglycemic wild-type mice, which in *Lrg1^-/-^* mice remained unchanged (Fig. 2E, fig. S4, D and E). To understand the potential functional impact of these early mild vascular changes, we applied an image-based computational modelling framework (34, 35) to predict blood dynamics and tissue oxygenation based on capillary diameter and spatial organization (fig. S4F). Simulations revealed that LRG1-mediated vascular remodeling in diabetes was associated with lower tissue oxygenation and higher hypoxia susceptibility (Fig. 2, F and G). This was directly linked to capillaries having smaller diameters (Fig. 2E) and a more sparse spatial organization (Fig. 2F) resulting in decreased blood flow rate (Fig. 2G), that was not compensated for by the subsequent increase in extraction coefficient (fig. S4G) induced by the longer red blood cell transit times. Additionally, hyperglycemic mice exhibited augmented basement membrane (BM) thickness (Fig. 2, H to J and fig. S4, H and I), a hallmark of DR, which may in turn impact EC shear-stress response and blood flow regulation (36), a feature that was not seen in *Lrg1*-deficient mice.

To test whether these changes impact neuronal function, we undertook electroretinography (ERG) to evaluate the electrical activity of the retina in response to light stimuli. In wild-type hyperglycemic mice (*Lrg1^+/+^* STZ, 6 month), we observed a decrease in b-wave amplitude, indicative of inner retinal dysfunction and consistent with previous reports (37, 38), whereas hyperglycemic *Lrg1*-deficient mice gave normal ERG responses (Fig. 2K and fig. S5). The b-wave response is associated with the activity of bipolar cells, which rely on the deep vascular plexus for oxygen and nutrients. Together, these findings demonstrate that LRG1 is required for the onset of hyperglycemia-induced early capillary dysfunction and provide evidence that LRG1-driven early vascular changes are sufficient to have a negative impact on retinal function.

### LRG1 activates a more contractile and fibrotic phenotype in retinal pericytes

**Fig. 3:**
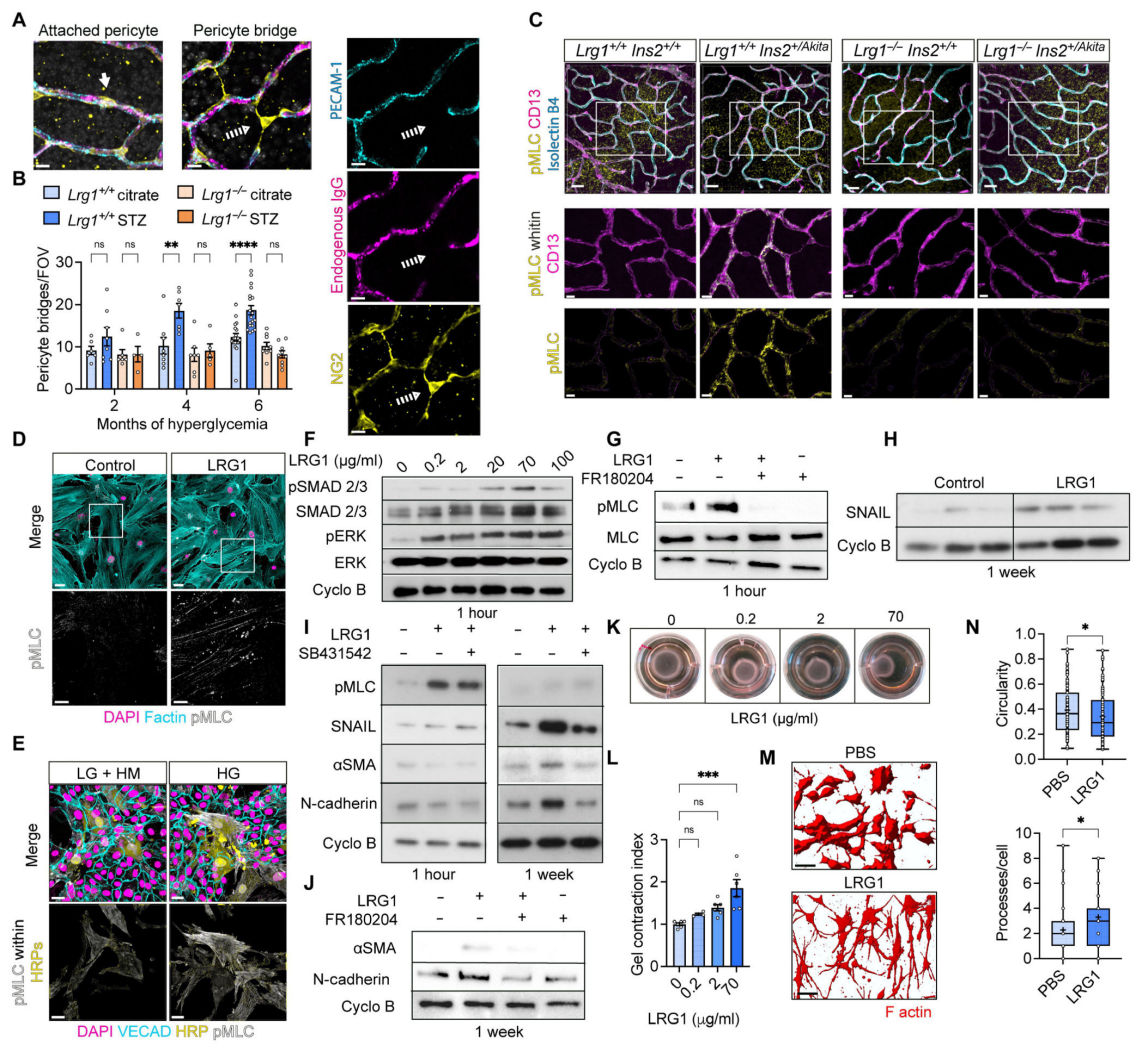
LRG1 promotes pericyte transition to contracted state. (**A**) Representative confocal images of a pericyte (NG2, yellow) attached to retinal capillaries (PECAM-1, cyan; vessel lumen endogenous IgG, magenta) and a PB. Attached pericyte (solid arrow), PB (dashed arrow). Single channel highlighting lack of overlap between the pericyte (NG2) and the vessel markers (PECAM-1 and IgG). Scale bar, 10 μm. (**B**) Pericyte bridges per field of view (fov) in the retinal deep vascular plexus at the time points indicated. n=6-19 mice per group, cumulative of 3 independent experiments. Fov is 581 x 581 μm as shown in fig. S6A. 2-way Anova; Tukey’s test for multiple comparisons. (**C**) Confocal images of retinal deep plexus of 6-month hyperglycemic mice and control littermates stained for vessel marker (isolectin B4, cyan), pericyte (CD13, magenta) and pMLC(yellow) (top images). Scale bar, 100 μm. pMLC signal within a CD13 mask (middle images). pMLC CD13-masked channel in isolation (bottom images). (**D**) Confocal images of HRPs treated with human recombinant LRG1 and stained for pMLC (white) and F actin (cyan). Scale bar, 50 μm. Images are representative of 3 independent experiments. (**E**) Confocal images of HRPs and HRECs co-cultured in low glucose (LG + HM)) or high glucose (HG) for 1 week. HRPs stained with fluorescent cell marker cmfda (yellow) prior to mixing. HRECs are VE-Cadherin^+^ (cyan), pMLC in white. pMLC signal within the HRP mask (bottom panels). Scale bar, 30 μm. Images are representative of 3 independent experiments. (**F**) HRPs treated for 1 hour with increasing concentrations of LRG1 and probed for pSMAD2/3, total SMAD2/3, pERK, total ERK and cyclophilin B (Cyclo B, loading control). Images are representative of 3 independent experiments quantified in fig. S7. (**G**) HRPstreated for 1 hour with LRG1 in the presence of the ERK inhibitor FR180204. Cell lysates probed for pMLC, total MLC and cyclophilin B (Cyclo B, loading control). Images are representative of 3 independent experiments quantified in fig. S8B. (**H**) HRPs treated for 1 week with LRG1 and probed for SNAIL and Cyclo B (loading control).(**I**) HRPs treated for 1 hour or 1 week with LRG1 in the presence of ALK5 inhibitor SB431542 and probed for pMLC, SNAIL, αSMA, N-Cadherin and Cyclo B. Images are representative of 3 independent experiments quantified in fig. S9D. (**J**) HRPs treated for 1 week with LRG1 in the presence of the ERK inhibitor FR180204 and probed for αSMA, N-Cadherin and Cyclo B. Images are representative of 3 independent experiments quantified in fig. S9G. (**K**) Representative photos of gels in a 24-well plate, containing a mix of HRPs and collagen I after overnight treatment with LRG1 at the concentrations indicated. (**L**) Contraction index is calculated as initial gel area divided by final area. n=4-6. 1-way Anova; Dunnet’s test for multiple comparisons. (**M**) Gels stained with phalloidin (F actin, red) to visualize HRP shape. Scale bar, 200 μm. (**N**) HRPs treated for 1 week with LRG1. The number of processes and cell circularity calculated for individual cells. Data points are cumulative of 3 independent experiments. All data points in bar charts represent mean and standard error of the mean. Whiskers in box plots extend to min and max values, box extends from the 25th to 75th percentiles and the line in the middle of the box is plotted at the median. P*<0.05; P**<0.01; P***<0.001; P****<0.0001.

To investigate whether the LRG1-driven vascular phenotype was associated with early pericyte dysfunction, we followed the time course of appearance of pericyte bridges (PBs) (39) in the retinal deep vascular plexus of hyperglycemic mice. PBs are pericytes that appear semi-detached from capillaries and have been shown previously to be an early pathological feature of DR (39, 40). Whereas diabetic wild-type mice exhibited an increase in PBs by 4 months, this did not occur in *Lrg1* knockout mice, indicating that LRG1 contributed to this pathological feature (Fig. 3, A and B and fig. S6A). Given the decrease in capillary diameter in 6-month hyperglycemic retinas, we investigated whether this could be linked to pericyte contraction and myosin activation (41, 42). In accordance with this, we observed an increase in the active phosphorylated form of myosin light chain (pMLC) in retinal pericytes of hyperglycemic wild-type mice, but not in *Lrg1*-knockout mice (Fig. 3C and fig. S6B). To explore whether LRG1 could directly mediate this effect, human retinal pericytes (HRPs) were treated with recombinant human LRG1 for 1 hour, resulting in the activation of MLC (Fig. 3D, fig. S6C and D). Similarly, when HRPs were co-cultured with HRECs under high glucose, HRPs showed more active MLC, supporting the idea that LRG1 released by ECs in hyperglycemia drives pericyte contraction (Fig. 3E and fig. S6E). Given that extracellular signal-regulated kinase 1 (ERK), a component of the non-canonical TGFβ signaling pathway, is a known driver of MLC phosphorylation (43) and that LRG1 has been shown to modulate TGFβ canonical and non-canonical signaling (11), we treated HRPs with increasing doses of LRG1 and observed that both non-canonical ERK and canonical small mothers against decapentaplegic 2/3 (SMAD2/3) pathways were activated (Fig. 3F, fig. S7). An ERK inhibitor (fig. S8A) demonstrated that LRG1-induced MLC phosphorylation is dependent on ERK (Fig. 3G and fig. S8B). Since TGFβ signaling can drive cell differentiation, we investigated the effects of longer conditioning (1 week) of HRPs with LRG1, which better reflects the chronic exposure of retinal pericytes to LRG1 in vivo. A proteomic screen suggested upregulation of the transcription factor SNAIL (fig. S9A), which was indeed increased in HRPs treated with LRG1 for 1 week (Fig. 3H and fig. S9B). This is consistent with LRG1 driving phenotypic changes in pericytes, as SNAIL is involved in driving mesenchymal transition (44), fibroblast activation and myofibroblast transdifferentiation (45). In accordance with this, we showed that myofibroblast-associated proteins such as α smooth muscle actin (αSMA), N-cadherin, vimentin, and fibronectin were all upregulated in HRPs treated with LRG1 for 1 week, indicating transition towards that lineage and that this was dependent on both canonical SMAD2/3 and non-canonical ERK signaling pathways (Fig. 3I, fig. S9, C to G). Indeed, using the activin receptor-like kinase 5 (ALK5) inhibitor SB431542 to restrict the SMAD2/3 pathway (fig. S9C), we showed that ERK but not ALK5 is required for the rapid MLC phosphorylation (Fig. 3I and fig. S9D), but both ALK5 and ERK inhibitors decreased αSMA and N-cadherin expression at 1 week, indicating the contribution of both pathways to this long-term effect (Fig. 3, I and J, fig. S9, D and G). As our data point toward LRG1 driving a contractile pericyte phenotype, we next investigated the impact of LRG1 conditioning on HRP contractility. HRPs were embedded in a collagen I-based gel overnight with increasing doses of LRG1, and gel contraction was quantified. The results showed that LRG1 increased HRP contractility (Fig. 3, K and L). The expression of F-actin in embedded HRPs revealed a more elongated and branched cell shape (Fig. 3, M and N) and the presence of stress fibers (fig. S9H), all consistent with an activated/contractile phenotype (46). Together these findings provide evidence that LRG1 modulates pericyte behavior, in part through activation of both canonical and non-canonical TGFβ signaling, promoting a more contractile and fibrotic phenotype.

### Blocking LRG1 prevents pericyte activation in vitro and attenuates early signs of DR in vivo

**Fig. 4:**
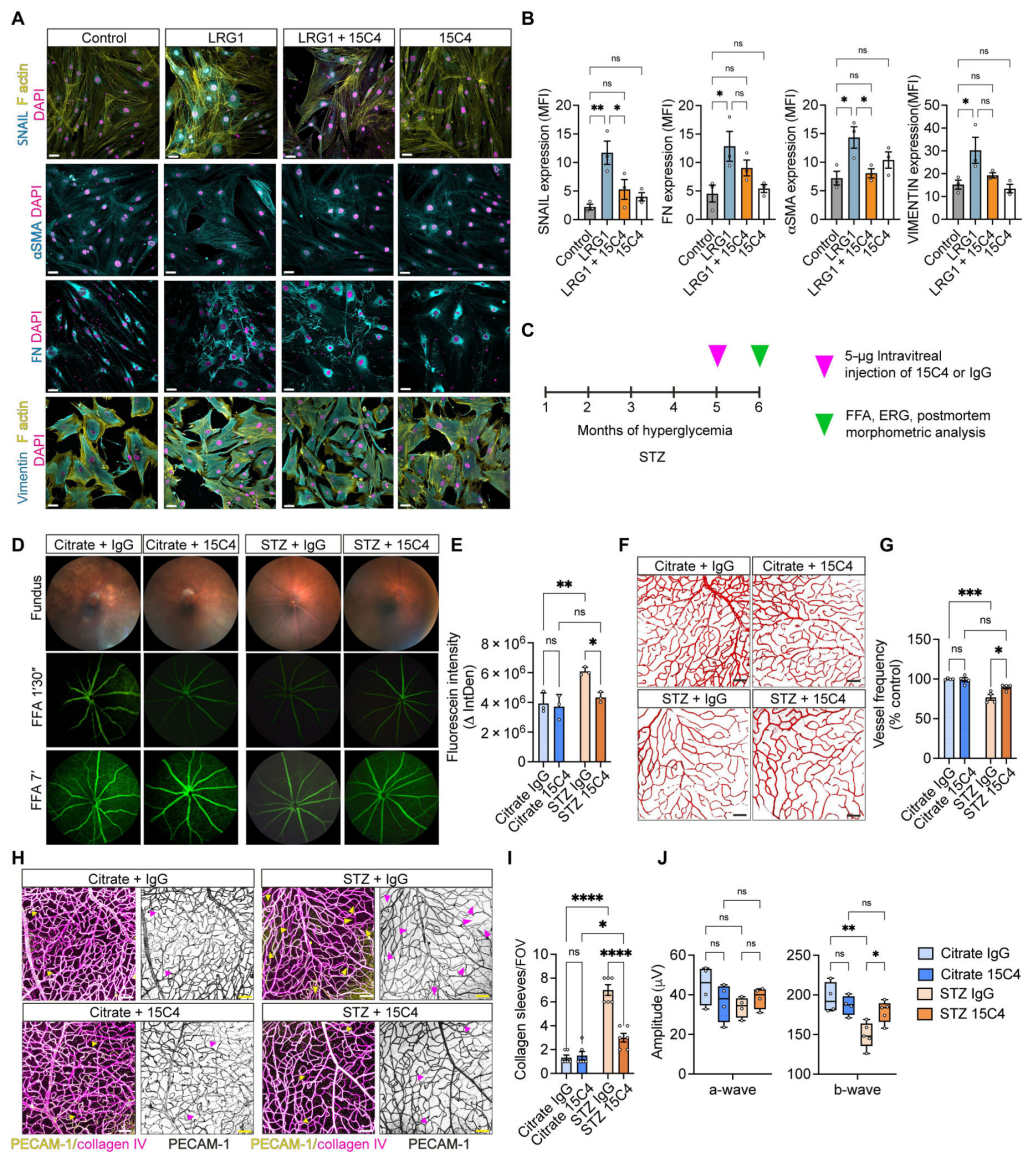
Blocking LRG1 prevents pericyte phenotypic switch and attenuates early signs of DR in vivo. (**A**) Confocal images and (**B**) mean fluorescence intensity (MFI) quantification of HRPs treated with 70 μg/ml recombinant human LRG1 (LRG1) and 500 μg/ml of 15C4 for 1 week. Cells stained for SNAIL, αSMA, FN, Vimentin (all in cyan) and F actin (yellow) and nuclei were visualized with DAPI (magenta). Images are representative of 3 independent experiments. Scale bar, 50 μm. 1-way Anova; Tukey’s test for multiple comparisons. (**C**) STZ mice with 5 months of hyperglycemia and controls were injected with 5 μg of 15C4 in one eye and 5 μg of IgG1 control in the contralateral eye and signs of diabetic retinopathy were evaluated 1 month thereafter (6 months of hyperglycemia in total). (**D**) On the top, representative fundus images from the 4 groups. Below, FFA angiograms of the same mice 90 sec and 7 min following subcutaneous fluorescein injection. (**E**) Leakage expressed as delta integrated density (ΔIntDen) of fluorescence between the two time points. n=3 mice per group. 2-way Anova; Tukey’s test for multiple comparisons. (**F**) Confocal images of the retinal deep plexus of the 4 groups at 6 months post-hyperglycemia. PECAM-1^+^ vasculature (red). Scale bar, 50 μm. (**G**) Vessel density expressed as the number of vessels crossing an arbitrary line, normalized to control (citrate, injected with IgG). n=4 mice per group. 2-way Anova; Tukey’s test for multiple comparisons. (**H**) Representative confocal images of retinas from the 4 groups. Arrows point to acellular capillaries (collagen sleeves), Collagen^+^ (magenta) and PECAM-1^-^ (yellow). (**I**) Collagen sleeves per fov quantified. Fov is 581 x 581 μm. n=6 mice per group. 2-way Anova; Tukey’s test for multiple comparisons. (**J**) ERG response after short dark adaptation in the 4 groups. n=4 mice per group. 1-way Anova; Tukey’s test for multiple comparisons. All data points in bar charts represent mean and standard error of the mean. Whiskers in box plots extend to min and max values, box extends from the 25th to 75th percentiles and the line in the middle of the box is plotted at the median. P*<0.05; P**<0.01; P***<0.001; P****<0.0001.

Having established that LRG1 affects pericyte behavior during the early stages of DR, we tested whether inhibiting LRG1 would prevent pericyte hypercontraction and therefore mitigate its contribution to vascular damage in the hyperglycemic retina. HRPs were cultured for 1 week in the presence of LRG1 with or without an LRG1-blocking antibody (15C4) (47). Protein analysis showed that treatment of pericytes with 15C4 prevented LRG1 mediated increase in the expression of SNAIL, αSMA, fibronectin, Collagen 1α1 and vimentin (Fig. 4, A and B and fig. S10, A and B). Having shown that 15C4 can block the effects of LRG1 on pericytes in culture, we next investigated whether it could prevent vascular dysfunction in early DR in vivo. We administered a 5 μg intravitreal injection of 15C4 to one eye and an equivalent dose of an isotype-matched control antibody to the contralateral eye of STZ mice after 5 months of hyperglycemia, along with citrate control mice (Fig. 4C). This timing corresponds to the onset of observable vascular dysfunction in these mice (Fig. 2). Analysis of retinal changes associated with DR, including hyperpermeability (Fig. 4, D and E), capillary density (Fig. 4, F and G), acellular capillaries (or collagen sleeves) and EC to pericyte ratio (Fig. 4, H and I and fig. S10 C and D) revealed that inhibiting LRG1 mitigates these early deleterious effects. Functional testing through ERG also revealed a rescue effect by 15C4 on the decline in b-wave amplitude in hyperglycemic mice (Fig. 4J). Comparable protective effects were also observed in Ins2Akita mice, where LRG1 blockade ameliorated the impaired ERG response induced by diabetes (fig. S10, F and G). These findings demonstrate that functional blockade of LRG1 with just a single dose of antibody ameliorates retinal dysfunction during the critical early steps of DR.

## Discussion

Using animal models that recapitulate early signs of DR, we have shown that *Lrg1* is induced in the retina before the prototypic vasoactive factor *Vegfa* and demonstrated that it disrupts retinal vascular function and density at this early stage by altering pericyte function (fig. S11). Computational modelling showed that this primary disturbance of the microvasculature is predicted to compromise blood flow and oxygen delivery to the retina. In mouse models, we observed that *Lrg1*-knockout protects mice from developing hyperglycemia-induced early vascular dysfunction and associated impairment of the ERG b-wave. This is in accordance with our modelling data, and with reports that b-wave anomalies in rodents are a sensitive indicator of retinal hypoxia (48). Such findings show that, in the retina, early vascular changes can tip the balance towards oxygen deprivation, especially in the outer plexiform layer where an oxygen watershed zone exists between the retinal superficial and deep circulations. Our data showed that a key priming event in the pathogenesis of DR is hyperglycemic induction of *Lrg1* gene expression in retinal ECs and that the vasculature is the major local source of LRG1 in the early diabetic retina. This is consistent with studies in other retinal disease models (11, 49) and in human disease (50), but does not rule out other cell types. Indeed, myofibroblasts (50) and microglia might contribute to intraocular LRG1 as the disease progresses and LRG1 may also enter the retina from the circulation through a compromised blood-retinal barrier. Further research employing EC- or other cell-specific LRG1 ablation strategies will be necessary to delineate the distinct contributions to the pathological effects observed in the diabetic retina at different disease stages.

The presence of EC-derived LRG1 in the retina has previously been shown to drive pathogenic neovascularization through an autocrine loop that mediates a switch in the TGFβ signaling pathway towards the pro-angiogenic ALK1-SMAD1/5 pathway (11). This mechanism is also mirrored in neovessels of diabetic kidney (51) and tumor (52). However, here, in the pre-neovascular stage of diabetic eye disease, LRG1 drives changes to the existing vasculature through paracrine signaling to pericytes. Contrary to ECs, and likely due to the presence of a different repertoire of TGFβRII co-receptors, LRG1 in pericytes hyperactivates TGFβ ALK5-SMAD2/3 signaling and the non-canonical ERK-pathway driving contraction and transition towards a more fibrotic phenotype. Pericytes are an essential component of the neurovascular unit and their intimate association with ECs is critical to the maintenance of vascular stability. Thus, any disturbance to pericyte function, believed to be a hallmark of early vascular damage in DR, is likely to compromise endothelial performance and overall vessel integrity. Our observations that the hyperglycemia-induced PBs, reported to be an early sign of diabetic vascular pathology (39), and phosphorylation of MLC are attenuated in the *Lrg1* knockout mouse provide evidence that LRG1 induction is an initiating event. Indeed, early changes in the contractile state of pericytes and the advent of vascular anomalies preceded *Vegfa* induction, showing that VEGF is not an initiating factor.

Pericytes are recognized as having contractile properties that can regulate capillary diameter and blood flow in the retina (53–55), but the chronic pericyte contraction we observed here may be a mechanism by which DR vascular pathology is initiated. This would be consistent with evidence of similar events in other organs. For instance, following an ischemic insult, coronary pericytes can contract and restrict capillary blood flow preventing re-flow (56). Similarly, in a murine model of brain ischemia, it was noted that, following contraction, pericytes die ‘in rigor’ and continue to restrict blood flow (57). It is possible that pericyte hyper-contraction and subsequent decreased blood flow will be followed by capillary collapse and EC death. Loss of ECs would then explain the PB formation, although we cannot rule out pericyte migration as an alternative or coexisting mechanism. Either way, deprivation of physical contact and crosstalk with ECs might result, in the long run, in pericyte apoptosis. This hypothesis agrees with the evidence of fewer retinal pericytes observed later in the disease (32, 33, 58). Importantly, and in agreement with our study, perfusion alterations in basal retinal blood flow have been observed in patients with diabetes, with retinal hypoperfusion often associating with the early stage of the disease. In addition to a basal alteration, light-evoked vasodilation, a process termed functional hyperemia, is also reduced in people with diabetes (59–63) and this can be observed in patients with diabetes with no or only mild signs of DR (59, 62).

This investigation establishes a mechanism of early initiating vascular dysfunction likely to be relevant to human diabetic eye disease, where LRG1 is found elevated in the plasma and vitreous (16, 18–20). Our findings provide the first direct evidence of the presence of LRG1 within the retinal parenchyma of individuals with diabetes. Future studies with larger cohorts will be essential to establish a robust correlation between retinal LRG1 expression, diabetes and DR progression. Such investigations will be critical for refining patient stratification strategies and informing the design of potential clinical trials aimed at targeting LRG1 in diabetic retinal disease.

Intravitreal administration of an LRG1-blocking antibody effectively reversed functional defects, raising the possibility of early therapeutic intervention to limit progression to the more devastating stages of the disease. The positive impact of LRG1 inhibition contrasts with studies showing that antibody blockade of VEGF in Ins2Akita mice does not improve visual transduction, rather it exacerbates the defect possibly because of the neuroprotective role of VEGF (64). Furthermore, the involvement of LRG1 in neovascularization (11) may also render LRG1 blockade a valid approach for the treatment of PDR(65).

This study has several limitations. The post-mortem retinal tissues lacked clinical metadata, including information on glycemic control, pharmacological interventions, retinopathy severity, ophthalmic imaging and age- and sex-matching. In addition, the supraphysiological glucose concentrations commonly used in vitro, as well as those observed in diabetic mouse plasma, may not represent the glycemic profiles of most individuals with diabetes undergoing insulin therapy. Finally, additional studies are warranted to confirm the vascular origin of increased LRG1 expression in human diabetic retina and validate our findings obtained in HRECs and in mice.

Limited understanding of the initiating pathophysiological events in DR have restricted the development of new therapeutic regimens aimed at slowing or halting progression to the later sight-threatening stages of the disease. In this study, we showed that hyperglycemia-induced LRG1 acts on pericytes to trigger early vascular anomalies, initiating retinal dysfunction. This new paradigm opens the possibility of early therapeutic intervention targeting LRG1 in patients who, using new modalities such as high-resolution wide-field imaging, have been identified as exhibiting early sub-clinical features.

## Materials And Methods

### Study design

The study aimed at investigating the role of LRG1 in the early vascular events of DR. Detailed information regarding the number of replicates, the statistical tests used, and the corresponding P values is provided in the figure legends. For in vivo experiments, the required number of animals was established using power analysis (alpha level set at 5% and power at 80%). Effect size was based on similar studies or small-scale pilot experiments. Exclusion criteria were pre-established where some animals developed a cataract or intra-ocular bleeding post intravitreal injection. Mice were randomly assigned to each group. Researchers were blind for the animal group during analysis. In vitro experiments were repeated 3 independent times.

### Animals and experimental design

Six to 8-week-old male C57BL/6J mice were obtained from Charles River and housed in a temperature-controlled environment with a 12-h light/dark cycle. Mice were fed standard chow and water ad libitum. The STZ model of T1D involves destruction of the insulin-producing beta cells in the pancreas, resulting in hyperglycaemia two weeks after induction. The STZ diabetic mouse model was established by intraperitoneal injection of STZ (Sigma) at a dose of 55 mg/kg body weight in tri-sodium citrate buffer for 5 consecutive days. Control animals received buffer alone. Body weight and glucose were measured every 2 weeks in the first month and at least monthly thereafter (fig. S4A and B). Ins2Akita mice harbor a mutation in the insulin 2 gene leading to decreased insulin production and hyperglycemia from two months of age and were obtained from the laboratory of David Attwell (UCL). Wild-type C57BL/6J females were crossed with heterozygous Ins2Akita (Ins2^+/Akita^) males. Litters were therefore composed of 50% hyperglycemic mice (Ins2^+/Akita^) and normoglycemic controls (Ins2^+/+^), of these only males were used for experiments as females develop milder hyperglycemia. Mice exhibited hyperglycemia by the first month of age. Body weights and glucose were measured at least monthly (fig. S4A and B). Mice were considered diabetic when fasting blood glucose exceeded 15 mmol/L. All procedures were performed in accordance with the UK Animals (Scientific Procedures) Act and the Animal Welfare and the Ethical Review Bodies of the UCL Institute of Ophthalmology.

### Western blotting

Cells were washed with PBS and lysed in RIPA buffer (Thermo Fisher Scientific) supplemented with protease and phosphatase inhibitors (Roche). Lysates were sonicated and centrifuged at 12,000 rpm for 15 min at 4°C to remove debris. The protein concentration of the supernatant was measured using the Pierce BCA Protein Assay Kit (Thermo Fisher Scientific). For plasma samples, blood was collected in lithium-heparin-coated microvettes and centrifuged at 4,000 rpm for 20 min at 4°C. Equal amounts of protein were then reduced and denatured using SDS-based loading buffer and reducing agent. Samples were separated by SDS-PAGE using 4-20% gradient gels (Bio-Rad) and transferred to PVDF membranes (Bio-Rad). Membranes were blocked in 5% non-fat dry milk or 5% BSA in PBS-T or TBS-T (0.1% Tween 20) for 1 hour at room temperature, and then incubated with primary antibodies against the protein of interest and loading control overnight at 4°C. After washing with PBS-T or TBS-T, membranes were incubated with horseradish peroxidase-conjugated secondary antibodies (DAKO) for 1 hour at room temperature. Protein bands were detected using enhanced chemiluminescence (ECL) reagent (GE Healthcare), visualized using the ChemiDoc MP Imaging System (Bio-Rad) and quantified using ImageJ software (NIH). All statistical analyses were performed using GraphPad Prism software.

### Solid phase sandwich ELISA Assay

Conditioned medium was collected from HRECs after 48 hours of treatment in EGM-2 medium (#CC-3162, Lonza) supplemented with 1% FBS. Cells were exposed either to 5.5 mM D-glucose with up to 25 mM D-mannitol (LG + HM, osmotic control) or to 25 mM D-glucose (HG, high glucose), with or without the NF-κB inhibitor BAY 11-7082 (1 μM; #78679, Cell Signaling). The medium was then passed through a 0.2-μm filter, and 1× Complete protease inhibitor cocktail (Roche #11697498001) was added. Human LRG1 was quantified using ELISA kit by IBL (#27769) according to manufacturer’s instructions.

### Chromatin immunoprecipitation assay

HRECs were cultivated in EGM-2 medium (#CC-3162, Lonza) with 1% FBS and either 5.5 mM D-glucose up to 25 mM with D-mannitol (low glucose, LG + HM, osmolalic control), or 25 mM of D-glucose (HG, high glucose). After 1, 24 and 48 hours 3 million cells were harvested and processed as described (66). Once obtained, the nuclear pellet was resuspended in ice-cold CHIP Buffer (25 mM Tris-HCl, 150 mM NaCl, 2 mM EDTA, 1% Triton X-100, 0.25% SDS, Protease inhibitor) and sonicated on ice 15 x 20 sec cycles at 14 micron amplitudes using a MSE (UK) Ltd Soniprep 150 to obtain 200-600 bp fragments. DNA-crosslinked proteins were immunoprecipitated (5% kept as input) using 5 μg of NF-κB/p65 (sc-8008, Santa Cruz) or control mouse IgG antibody (M8695, Sigma) and protein G magnetic beads (Dynabeads, Thermo Fisher) at 4°C for 4 hours. DNA was then purified using PureLink Quick PCR purification Kit (Invitrogen). Immunoprecipitated DNA and input DNA were used as a template for real-time PCR (see method below), with the following primers obtained from (25): RELA1 (FOR-CCAGGAATAGTGCCTTGCAAA; REV-GCCTTATACCTGCCTGGAC TGG), RELA 2 (FOR-GCACACACACACACACCC CTA; REV-GCTCACTGCAGCCTCTGAA). Samples Ct values were normalised over the Ct of their respective input, then over the negative control and finally expressed as fold enrichment over their experimental controls (HRECs in low glucose).

### In situ RNA hybridization

Retinas were dissected in ice-cold PBS, fixed in 10% formalin overnight, and then mounted on a glass slide with the ganglion cell layer facing up and allowed to air dry for 30’. RNA was detected using the RNAscope Multiplex Fluorescent Reagent Kit 2 (Advanced Cell Diagnostics). Briefly, after air drying, retinas were treated with H_2_O_2_ to quench endogenous peroxidase activity, and then incubated with protease plus (ACD) target-specific probes, followed by a series of amplification steps (each for 30’ at 40°C). Each amplification step was followed by washing steps to remove unbound probes. Finally, the retinas were mounted with Prolong Gold (Thermo Fisher Scientific).

### Cell culture

HRPs at passage 3 were purchased from Cell Systems (#ACBRI 183 V) and cultured in DMEM (#D6546, Sigma-Aldrich) supplemented with 10% fetal bovine serum (FBS) and antibiotics (100 U/mL penicillin and 100 μg/mL streptomycin) at 37°C in a humidified atmosphere of 5% CO_2_. The medium was changed every 2-3 days, and the cells were passaged at 80% confluence using trypsin-EDTA (0.05%). The cells were used for experiments at passages 5-7. Human retinal ECs at passage 3 were purchased from Cell Systems (#ACBRI 181 V) and cultured in complete endothelial cell growth medium with EC supplement (#H1168, Cell Systems), 10% fetal bovine serum (FBS) and antibiotics (100 U/mL penicillin and 100 μg/mL streptomycin) at 37°C in a humidified atmosphere of 5% CO_2_. The medium was changed every 2-3 days, and the cells were passaged at 80% confluence using trypsin-EDTA (0.05%) and used for experiments at passages 5-7. Both cell types were cultured on supports pre-coated with 2% gelatin-based coating solution (Cell Systems). Unless stated otherwise, human recombinant LRG1 was used at a concentration of 70 μg/ml. Alk-5 inhibitor SB431542 was used at 26 mM in serum-free DMEM (#301836-41-9, Sigma-Aldrich) and Erk1/2 inhibitor FR180204 was used at 50 μM in serum-free DMEM (#15544-5mg-CAY, Cayman Chemical). For high glucose experiments, human retinal ECs were cultivated in EGM-2 medium (#CC-3162, Lonza) with 1% FBS and either 5.5 mM D-glucose (low glucose), 5.5 mM D-glucose up to 25 mM with D-mannitol (high mannitol, osmolalic control), or 25 mM of D-glucose (high glucose). STAT3 inhibitor AG490 was used at 50 μM (#658411, Sigma-Aldrich), NF-κB inhibitor BAY 11-7082 was used at 1 μM (#78679, Cell Signaling).

### Whole-mount retina immunofluorescence staining and imaging

Eyes were enucleated and the retinas were carefully isolated under a dissecting microscope. The retinas were then fixed in 4% paraformaldehyde or methanol for 20 min at room temperature and washed with PBS. After washing, retinas were permeabilized with 3% Triton X-100, 1% Tween-20 in PBS for 2 hours at room temperature. Non-specific binding was blocked by incubating the retinas in 5% normal donkey serum in PBS for 2 hours at room temperature. Retinas were then incubated with primary antibodies against the target proteins (table S2) diluted in 5% normal donkey serum in PBS Tween-20 at 4°C overnight. Retinas were washed three times with PBS Tween-20 and then incubated with fluorescently labelled secondary antibodies (table S2) diluted in 5% normal donkey serum in PBS Tween-20 for 2 hours at room temperature. Finally, retinas were washed three times with PBS and mounted on glass slides with Prolong Gold (Thermo Fisher Scientific). Images of murine retinas were acquired using a confocal microscope (Leica SP8, Stellaris 5 or Stellaris 8 with white-laser capability). Z-stack images were taken at a resolution of 1024x1024x17 pixels with a voxel size of 0.568x0.568x1.04 μm, lens power 20, numerical aperture 0.75.

### Fluorescein fundus angiography

Mice were anesthetized with a mixture of Narketan and Dormitor, their pupils were dilated with 1% tropicamide and eyes kept moist with Viscotears. Mice were placed on a heated platform and injected subcutaneously in the neck fold with 0.1 ml of 2% sodium fluorescein solution (Sigma-Aldrich). Fluorescence images were acquired using a fundus camera (Phoenix Micron III) at 90 sec and 7 min after injection. Images were processed and analysed using ImageJ software (NIH) to quantify the area of integrated density of fluorescence at the two time points. Results are shown as difference in these values between the 7 min and 90 sec time points.

### Electroretinography

ERG recordings were performed on hyperglycemic and control mice after 6 months of hyperglycemia. Mice were dark-adapted for 30 min and then anesthetized with a mixture of Narketan and Dormitor, and their pupils were dilated with 1% tropicamide and 2.5% phenylephrine hydrochloride. ERG recordings were performed using the Diagnosys LLC Celeris system. ERG responses were evoked by brief flashes of light stimuli (5 ms duration and 1.03Hz frequency) at different light intensities ranging from 0.005 to 50 (P) cd.s/m^2^. The amplitudes of the a- and b-waves of the ERG response were measured and analyzed using the Espion software. The a-wave amplitude was measured from the baseline to the first negative trough, whereas the b-wave amplitude was measured from the a-wave trough to the peak of the positive wave. The data were analyzed using GraphPad Prism software.

### Transmission electron microscopy

Mouse eyes were fixed in 2% paraformaldehyde/2% glutaraldehyde in 0.15 M cacodylate buffer pH 7.4 for 2 hours at room temperature. Subsequently, eyes were incubated in 1% osmium tetroxide/1% potassium ferrocyanide for 1 hour before incubating in 1% aqueous thiocarbohydrazide for 20 min, and 2% aqueous osmium tetroxide for 30 min at room temperature. Additional en bloc staining was performed by incubating the eyes in undiluted UA-Zero (Agar Scientific) for 2 hours at room temperature in the dark. The eyes were dehydrated using increasing concentrations of ethanol and propylene oxide followed by infiltration with 1:1 propylene oxide:TAAB812 resin (TAAB) overnight at room temperature. The eyes were embedded by incubating in TAAB812 overnight at 60°C. ~100 nm thick sections were cut and imaged using a JEOL 1400+ TEM equipped with an Advanced Microscopy Technologies (AMT) XR16 charge-coupled device camera.

### Vasculature segmentation

Vascular segmentation was performed using Imaris software. Briefly, 3 Z-stack images of the PECAM-1^+^ deep vascular plexus were taken at 20X magnification in areas equidistant from the optic nerve and the periphery of the flat-mounted retina. Based on the PECAM-1 signal, an Imaris surface was created, masked and segmented using Imaris filament function. The same surface and filament creation parameters were applied to all images (n=6-9 per group). (Batched parameters are detailed in the Supplementary Methods).

### Simulations

Vascular networks were exported from Imaris to Matlab as.hoc files. Blood flow and tissue oxygenation were modelled using a pore-network approach coupled with Green’s functions using the skeletons of the microvascular networks. Model predictions were validated against in vivo measures of blood flow rates and intravascular oxygen concentration obtained from the literature for the healthy control group. Simulations were run for every network of each group and integral quantities were derived, namely the total flow rate perfusing the network, the extraction coefficient (the mass fraction of oxygen crossing the capillary walls) and the hypoxia susceptibility (a summary statistics for tissue oxygenation). Model formulation, validation and integral quantities are presented in detail in the Supplementary Methods.

### Contraction assay

Collagen I gel was prepared by mixing 1 mg/ml type I collagen (Corning) with 1x E4 medium and neutralized with 1N NaOH. Human retinal pericytes (Cell Systems) were trypsinized, resuspended in the collagen I gel, compounds added to the mix and plated in a non-TC-treated 24-well plate at a density of 3 × 10^5^ cells/ml. After the gel was allowed to polymerize for 30 min at RT, 500 μl of pericyte medium (Cell Systems) was added to the wells. The plates were incubated for 24 hours at 37°C and the diameter of the collagen I gel was measured using ImageJ software (NIH). Results are shown as contraction index, initial area divided by final area of collagen/cell mix. All statistical analyses were performed using GraphPad Prism software.

### Cell circularity analysis

HRP were treated for 1 week with 0-70 μg/ml human LRG1. Cell circularity was calculated as 4π(area/perimeter^2^) using Fiji Image J. A circularity value of 1.0 indicates a perfect circle. As the value approaches 0.0, it indicates an increasingly elongated polygon.

### Cell immunofluorescence staining and imaging

Cells were seeded onto glass 4- or 8-well chambers (Ibidi) pre-coated with 2% gelatin-coating solution (Cell Systems). Cells were fixed with 4% paraformaldehyde for 15 min at room temperature and permeabilized with 0.2% Triton X-100 for 5 min. After blocking with 1% BSA for 30 min, the cells were incubated with primary antibodies (table S2) against the protein of interest overnight at 4°C. The following day, cells were washed with PBS and incubated with Alexa Fluor-conjugated secondary antibodies (Thermo Fisher Scientific) for 1 hour at room temperature. Nuclei were stained with 4’,6-diamidino-2-phenylindole (DAPI, Thermo Fisher Scientific) for 5 min. Coverslips were mounted with Prolong Gold mounting medium (Thermo Fisher Scientific) and images were acquired using a confocal microscope (Leica Stellaris 5). Z-stack images were taken at a resolution of 1024x1024x12 pixels with a voxel size of 0.568x0.568x0.685 μm, lens power 20, numerical aperture 0.75 or lens power 63, numerical aperture 1.4, voxel size 0.180x0180x0.299 μm.

### Processing, staining and imaging of human retinas

Post-mortem globes from donors (table S1) were obtained through NHSBT under ethics approval (20/SW/0031-2022ETR84) and fixed in 4% PFA for 48 hours, before dissection to isolate post-equatorial retina, re-fixation in 1:4 Cytofix (BD Biosciences) overnight, then cryopreservation in 30% sucrose. Following embedding in OCT medium (Fisher Scientific), 15 μm cryosections were blocked and permeabilised for 1 hour in 1X perm/wash (BD biosciences), 1:100 Fc block (BD Pharmingen), 5% normal donkey serum. Primary antibodies (table S2) were then incubated over-night in a humified chamber at 4°C. Following washes in PBS, sections were incubated with secondary antibodies 1:400 (table S2) for 3 hours at room temperature, washed in PBS and mounted with Prolong Gold mounting medium (Thermo Fisher Scientific). Images were acquired using a confocal microscope (Leica Stellaris 8). Z-stack images were taken at a resolution of 1024x1024x35 pixels with a voxel size of 0.284x0.284x0.346 μm, using a 40x/1.30 HC PL APO objective.

### Statistical analysis

All analyses were blinded. Statistical analysis was performed using Graphpad Prism version 10.0 for Mac (GraphPad Software). Student t-test was used when two groups where compared, one-way ANOVA when one variable amongst multiple groups was considered and two-way ANOVA when two variables were considered. Definition of center, exact n, error bars and post-hoc analysis tests used for each experiment are indicated in the figure legends.

## Supplementary Material

Supplemental Material

Supplemental Material Reference

## Data Availability

All data associated with this study are in the paper or supplementary materials. Parameters and formulas adopted for the retinal vasculature segmentation and the oxygen/flow analysis are contained in the Supplementary Materials and Methods. Use of 15C4 is regulated by a Material Transfer Agreement (MTA). Code is available on Zenodo at https://zenodo.org/records/17211122

## One Sentence Summary

LRG1-driven microvascular dysfunction triggers early diabetic retinopathy, highlighting a potential therapeutic target.

## Editor’s summary

Diabetic retinopathy (DR) is common and can lead to visual impairment and blindness. Retinal vascular changes can occur early in disease, but mechanisms linking disease initiation and vascular changes are not well understood. De Rossi *et al*. found that leucine-rich *α*-2-glycoprotein 1 (LRG1) initiated microvascular pathology, including capillary narrowing and thickened basement membranes, early in mouse models of DR. Computational modeling revealed these early changes could impair oxygen delivery to the retina, which could result in defects in vision. Genetically deleting *Lrg1* or blocking LRG1 function with an antibody rescued disease phenotypes in the mouse models. Together, these results show that LRG1 is an early trigger of retinal dysfunction in DR, and that LRG1 could be a target for preventing the progression of DR. —Brandon Berry
