# Supplementary material for: Leucine-rich α-2-glycoprotein 1 initiates the onset of diabetic retinopathy in mice
Source: Sci Transl Med. Author manuscript; Available in PMC 2025 Dec 8. (PMC7618449; doi:10.1126/scitranslmed.adn6047)
Supplement: Supplemental Material [file EMS209684-supplement-Supplemental_Material.pdf]

Supplementary Materials

Materials and Methods

Retinal vascular segmentation using Bitplane Imaris 10.01.1. In this supplementary method we describe the surface and filament parameters applied to the image processing software Bitplane Imaris to segment the deep plexus of murine retinal vasculature immunostained for PECAM-1.

Surfaces Creation Parameters
  Format Version: 9.8
  Enable Region Of Interest: false
  Process Entire Image: false
  Enable Region Growing: false
  Enable Tracking: false
  Enable Classify: false
  Source Image Index: 0
  Source Channel Index: 1
  Enable Smooth: true
  Surface Grain Size: 1
  Enable Eliminate Background: true
  Diameter Of Largest Sphere: 8
  Enable Automatic Threshold: false
  Manual Threshold Value: 13.6123
  Active Threshold: true
  Enable Automatic Threshold B: true
  Manual Threshold Value B: 153.82
  Active Threshold B: false
  Region Growing Estimated Diameter: 12
  Region Growing Background Subtraction: true
  Enable Shortest Distance: false
  Surfaces Base Color: 0.8 0 0
  Track Base Color: 1 1 1
  Region Of Interest Container
  Region Growing Filter
    Statistics Filter
      Lower Threshold Enable: true
      Lower Threshold Manual: false
      Lower Threshold Manual Init To Auto: false
      Lower Threshold Manual Value: 21.0229
      Upper Threshold Enable: false
      Upper Threshold Manual: false
      Upper Threshold Manual Init To Auto: false
      Upper Threshold Manual Value: 0.594056
      Select High: true
      Manual Threshold: false
      Manual Threshold Value: 21.023
      Init Manual Threshold To Auto: false
      Statistics Value Type
        Name: Quality
        Unit:
        Factors: 0
  Surfaces Filter
    Statistics Filter

Lower Threshold Enable: true
Lower Threshold Manual: true
Lower Threshold Manual Init To Auto: false
Lower Threshold Manual Value: 3928.1
Upper Threshold Enable: false
Upper Threshold Manual: false
Upper Threshold Manual Init To Auto: false
Upper Threshold Manual Value: 4.6861e+07
Select High: true
Manual Threshold: true
Manual Threshold Value: 3928.100
Init Manual Threshold To Auto: false
Statistics Value Type
Name: Number of Voxels
Unit:
Factors: 1
Factor Name0: Image
Factor Level0: Image 1
Statistics Filter
Lower Threshold Enable: true
Lower Threshold Manual: true
Lower Threshold Manual Init To Auto: false
Lower Threshold Manual Value: 1500
Upper Threshold Enable: false
Upper Threshold Manual: false
Upper Threshold Manual Init To Auto: false
Upper Threshold Manual Value: 4.6861e+07
Select High: true
Manual Threshold: true
Manual Threshold Value: 1500.000
Init Manual Threshold To Auto: false
Statistics Value Type
Name: Area
Unit: um^2
Factors: 0
Object Tracking Algorithm Parameters
Track Algo Name: Autoregressive Motion
Fill Gap Enable: false
Reference Frames Id: 0
Object Tracking Algorithm Linear Asignment
Max Gap Size: 3
Max Distance: -1
Track Filter
Statistics Filter
Lower Threshold Enable: true
Lower Threshold Manual: true
Lower Threshold Manual Init To Auto: false
Lower Threshold Manual Value: 2.5
Upper Threshold Enable: false
Upper Threshold Manual: false
Upper Threshold Manual Init To Auto: true
Upper Threshold Manual Value: 1
Select High: true
Manual Threshold: true
Manual Threshold Value: 2.500

Init Manual Threshold To Auto: false
Statistics Value Type
Name: Track Duration Steps
Unit:
Factors: 0
Surfaces Classification Parameters
Category: Surface
Surfaces Event Parameters
Category: Surface

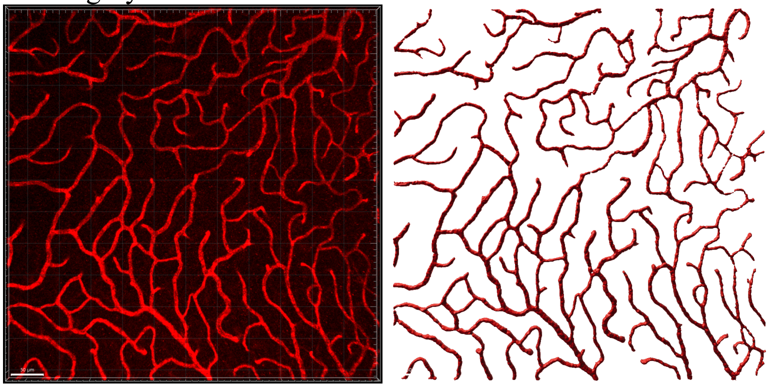

Original image, CD31 (red), on the left. Processed Surface on the right.

Filament Create Parameters Auto Path New
Format Version: 8.0
Step: eAlgorithm
Enable Regions Of Interest: false
Enable Regions Of Interest All Time Points: false
Processing Type: eAllTimePoint
Algorithm Type: eAutopathNewWithLoop
Source Image Index: 0
Source Channel Index: 0
Build All Time Points: true
Track: false
Track Dendrite: true
Track Spine: true
Track Graph Max Dist: -1
Track Graph Gaps Close: true
Track Graph Gaps Size: 2
Segment Branch Points Track Enabled: true
Segment Branch Points Max Distance: -1
Segment Branch Points Gap Close: false
Segment Branch Points Gap Size: 2
Segment Terminal Points Track Enabled: true
Segment Terminal Points Max Distance: -1
Segment Terminal Points Gap Close: false
Segment Terminal Points Gap Size: 2
Spine Attachment Points Track Enabled: true
Spine Attachment Points Max Distance: -1
Spine Attachment Points Gap Close: false
Spine Attachment Points Gap Size: 2
Spine Branch Points Track Enabled: true
Spine Branch Points Max Distance: -1
Spine Branch Points Gap Close: false
Spine Branch Points Gap Size: 2
Spine Terminal Points Track Enabled: true

Spine Terminal Points Max Distance: -1
Spine Terminal Points Gap Close: false
Spine Terminal Points Gap Size: 2
Enable Shortest Distance: false
Time Point: 0
Build All Time Points Enable: false
Calculate Soma Model Enable: false
Render Soma Model Enable: true
Dendrite Seed Point Remove Enable: true
Dendrite Seed Point Remove Diameter: 20
Dendrite Source Channel Index: 3
Dendrite Start Point Enable: false
Dendrite Start Point Diameter: 10
Dendrite Seed Point Diameter: 1.63508
Dendrite Seed Point Diameter Max Enable: true
Dendrite Seed Point Diameter Max: 28.9664
Dendrite Start Point Threshold Low Automatic Enable: true
Dendrite Start Point Threshold Low Manual: 0
Dendrite Start Point Threshold High Automatic Enable: true
Dendrite Start Point Threshold High Manual: 0
Dendrite Seed Point Threshold Automatic Enable: false
Dendrite Seed Point Threshold Manual: 31.2677
Dendrite Segment Radius Filter Factor: 3
Dendrite Seed Point Classification Enable: true
Dendrite Remove Outlier Enable: false
Dendrite Remove Outlier Filter Diameter: 1
Dendrite Remove Outlier Background Subtract Enable: false
Dendrite Remove Outlier Threshold Automatic Enable: true
Dendrite Remove Outlier Threshold Manual: 0
Dendrite Remove Outlier Algorithm Max Gap Length Enable: true
Dendrite Remove Outlier Algorithm Max Gap Length: 0
Dendrite Remove Outlier Algorithm Max Gap Ratio: 0.2
Dendrite Calculate Diameter Threshold Automatic Enable: true
Dendrite Calculate Diameter Manual: 3
Dendrite Caluclate Diameter Distance Map Enable: true
Spine Source Channel Index: 0
Spine Detect Enable: false
Spine Seed Point Diameter: 1
Spine Max Length: 2
Spine Branch Enable: false
Spine Seed Point Threshold Automatic Enable: true
Spine Seed Point Threshold Manual: 0
Spine Seed Point Classification Enable: true
Spine Calculate Diameter Threshold Automatic Enable: true
Spine Calculate Diameter Manual: 3
Spine Calculate Diameter Distance Map Enable: true
Fast Marching Stop Ratio: 0.5
Tree Segment Classification Enable: true
Segment Gap Max Length: 0
Tree Segment Gap Max Length Enable: false
Network Segment Gap Max Length: 0
Region Of Interest Container
Dendrite Seed Point Classification Parameters
  Category: SegmentSeed
  C Object Group Parameters

Name: Filter
Category: Spot
Classification Type: MachineLearning
Display: true
C Objects Inputs
  Objects Input Type: AllObjects
  Input Group Name: Group
  Input Class Name: Class
  Input Group Index: -1
  Input Class Index: -1
C Objects Class Parameters0
  Name: Keep
  Color: 0 1 1
C Objects Class Parameters1
  Name: Discard
  Color: 1 0 0
C Objects Classify Filter1 D
  Statistics Value Type
    Name: Area
    Unit: um^2
    Factors: 1
    Factor Name0: Category
    Factor Level0: Spot
  C Threshold Value
    Class Name: 0
    Threshold Low: -3.40282e+38
    Threshold High: 26.3597
  C Threshold Value
    Class Name: 1
    Threshold Low: 26.3597
    Threshold High: 3.40282e+38
C Objects Classify Filter2 D
  Statistics Value Type
    Name: Area
    Unit: um^2
    Factors: 1
    Factor Name0: Category
    Factor Level0: Spot
  Statistics Value Type
    Name: Average Distance To 5 Nearest Neighbours
    Unit: um
    Factors: 1
    Factor Name0: Category
    Factor Level0: Spot
  C Area Value
    Class Name: 0
    Area Min X: 0
    Area Min Y: 0
    Area Max X: 0
    Area Max Y: 0
  C Area Value
    Class Name: 1
    Area Min X: 0
    Area Min Y: 0
    Area Max X: 0

```
        Area Max Y: 0
      C Objects Classify R F
Spine Seed Point Classification Parameters
  Category: SpineSeed
Segments Classification Parameters
  Category: Segment
  C Object Group Parameters
    Name: Filter
    Category: Segment
    Classification Type: MachineLearning
    Display: true
    C Objects Inputs
      Objects Input Type: AllObjects
      Input Group Name: Group
      Input Class Name: Class
      Input Group Index: -1
      Input Class Index: -1
    C Objects Class Parameters0
      Name: Keep
      Color: 0 1 1
    C Objects Class Parameters1
      Name: Discard
      Color: 1 0 0
    C Objects Classify Filter1 D
      Statistics Value Type
        Name: HGD
        Unit:
        Factors: 2
        Factor Name0: Bin
        Factor Level0: 1
        Factor Name1: Category
        Factor Level1: Segment
      C Threshold Value
        Class Name: 0
        Threshold Low: -3.40282e+38
        Threshold High: 0.311366
      C Threshold Value
        Class Name: 1
        Threshold Low: 0.311366
        Threshold High: 3.40282e+38
    C Objects Classify Filter2 D
      Statistics Value Type
        Name: HGD
        Unit:
        Factors: 2
        Factor Name0: Bin
        Factor Level0: 1
        Factor Name1: Category
        Factor Level1: Segment
      Statistics Value Type
        Name: HGD
        Unit:
        Factors: 2
        Factor Name0: Bin
        Factor Level0: 3
```

Factor Name1: Category
Factor Level1: Segment
C Area Value
Class Name: 0
Area Min X: 0
Area Min Y: 0
Area Max X: 0
Area Max Y: 0
C Area Value
Class Name: 1
Area Min X: 0
Area Min Y: 0
Area Max X: 0
Area Max Y: 0
C Objects Classify R F
Network Terminal Segment Filter

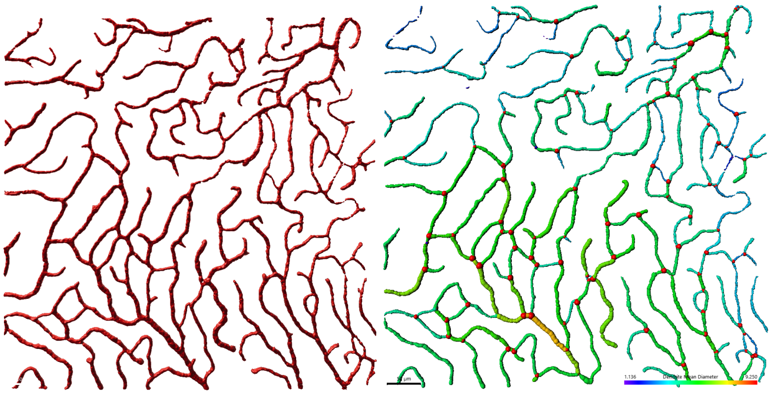

Processed Surface (red) on the left. Processed Filament network (color coded for diameter length, branch points in red) on the right.

Simulations of retina oxygenation. In this supplementary material we present the mathematical model at the basis of the simulations presented in the main manuscript, including the constitutive equations and boundary conditions (Section A) the model parameters (Section B), the model validation (Section C) and the integral quantities used in the main manuscript (Section D).

Section A. Model derivation

Blood flow. We model blood flow in microvascular networks using a well-established pore-network approach (*67-71*). Briefly, the microvascular network is associated with a graph, where each vessel is a segment, and each bifurcation is a node (fig. S4F). Blood flow conservation is then imposed at every node

$$\sum_{j \in \mathcal{V}(i)} Q_{ij} = 0, \quad \text{(a)}$$

where i and j are two neighbouring nodes, i.e. the start and end nodes of segment $\{ij\}$, where Q_{ij} is the blood flow rate associated with the segment $\{ij\}$, and where $\mathcal{V}(i)$ is the set of nodes neighbouring node i. Momentum conservation is then imposed between neighbouring nodes assuming no leakage so that

$$Q_{ij} = G_{ij}(P_i - P_j), \quad \text{(b)}$$

where P_i and P_j are the pressures associated with nodes i and j respectively and G_{ij} is the conductance of segment $\{ij\}$ defined as

$$G_{ij} = \frac{\pi d_{ij}^4}{128\mu_{ij}(H_{d,ij}, d_{ij})L_{ij}}, \quad \text{(c)}$$

where d_{ij} is the diameter and L_{ij} the length of the segment respectively and μ_{ij} the effective viscosity of the blood. The blood is modelled as a monophasic non-Newtonian fluid, so that μ_{ij} depends non-linearly on the discharge haematocrit $H_{d,ij}$, i.e., the local presence of red blood cells, using an established semi-empirical law (*72*).

Indeed, red blood cells distribute unevenly at diverging bifurcations (i.e., nodes with 1 inlet segment and 2 outlet segments) due to phase separation effects. Following fig. S4F labelling, this leads for the two outlet segments to

$$H_{d,ij} = FQE_{ij} H_{d,ik} \frac{Q_{ik}}{Q_{ij}}, \quad \text{(d)}$$

$$H_{d,il} = FQE_{il} H_{d,ik} \frac{Q_{ik}}{Q_{il}}, \quad \text{(e)}$$

where FQE_{ij} and $FQE_{il} = 1 - FQE_{ij}$ are the fraction of red blood cell flow going into segment $\{ij\}$ and $\{il\}$ respectively and are determined using another established semi-empirical relationship (*73*). Finally, red blood cell flow rate conservation is imposed at the remaining nodes so that

$$\sum_{j \in \mathcal{V}(i)} H_{d,ij} Q_{ij} = 0. \quad \text{(f)}$$

Taken together, equations (a) to (f) form a non-linear problem that is solved iteratively using the boundary conditions presented in Section A3.

Oxygen transport. Oxygen transport in the tissue is modelled using a diffusion-reaction equation, with oxygen metabolism modelled following the well-established Michaelis Menten kinetics law (*69-71, 74, 75*)

$$D_T \nabla^2 C_T = M_{\max} \frac{C_T}{C_{1/2} + C_T}, \tag{g}$$

where C_T represents the local oxygen concentration, D_T the diffusion coefficient of oxygen in tissue, $M_{\max}$ the maximum oxygen metabolic rate and $C_{1/2}$ the concentration for which oxygen metabolic rate is half its maximum value. We note that the concentration of oxygen in the tissue can also be expressed as a function of the partial pressure $C_T = \alpha_T P_{O_2,T}$, with α_T the effective solubility of oxygen in the tissue (Table S3). We use the established Green's function approach (*69-71*) to transform equation (g) into a distribution of sources and sinks

$$C_T(\boldsymbol{x}) = \iint_{S_N} \mathfrak{G}(\boldsymbol{x}, \boldsymbol{x}') q(\boldsymbol{x}') d\sigma(\boldsymbol{x}') - \iiint_{V_T} \mathfrak{G}(\boldsymbol{x}, \boldsymbol{x}') M_{\max} \frac{C_T(\boldsymbol{x}')}{C_{1/2} + C_T(\boldsymbol{x}')} dV(\boldsymbol{x}'), \tag{h}$$

where q represents the oxygen flux per surface area between vessel and tissue, $d\sigma$ the elementary vessel lateral surface, S_N the union of all lateral surfaces of segments forming the network, V_T the volume of tissue and dV the elementary volume of tissue. $\mathfrak{G}$ represents the Green's function associated with three-dimensional diffusion in an infinite domain

$$\mathfrak{G}(\boldsymbol{x}, \boldsymbol{x}') = \frac{1}{4\pi D_T |\boldsymbol{x} - \boldsymbol{x}'|}, \tag{i}$$

where $\boldsymbol{x}$ represents the local position and $\boldsymbol{x}'$ the position of the source or sink. Intravascular oxygen transport is then modelled using an advection-diffusion-reaction equation along the centreline of each segment, assuming only diffusive exchange with the tissue so that

$$\partial_z \phi = -K_{\text{eff}} S (C_V - C_T), \tag{j}$$

where C_V is the average intravascular oxygen concentration, z is the position along the segment centreline and ϕ is the mass flux going through a segment cross-section defined as

$$\phi = Q_{ij}\left(H_{d,ij} C_0 \Psi_{O_2} + C_V\right) - D_V \partial_z C_V S, \tag{k}$$

where Ψ_{O_2} represents the haemoglobin saturation in oxygen (i.e., the oxygen stored in red blood cells), C_0 the oxygen binding capacity of red blood cells, D_V the diffusion coefficient of oxygen in the blood and S the cross-section area of the segment. Further, we assume Ψ_{O_2} follows Hill's law so that

$$\Psi_{O_2} = \frac{C_V^n}{C_V^n + C_{50}^n}. \tag{l}$$

Finally, K_{eff} in equation (j) is the effective exchange coefficient (*76*), which is defined as

$$K_{\text{eff}} = \frac{K}{1 + \frac{Kd}{8 D_V}}, \tag{m}$$

where K is the segment wall permeability, estimated as $K \approx \frac{D_T}{e}$ where e represents the thickness of the segment wall, here taken as the thickness of the endothelium. We note that the intravascular concentration of oxygen can also be expressed as the partial pressure of oxygen, i.e., $C_V = \alpha_V P_{O_2,V}$, with α_V the oxygen solubility in the blood. Enforcing mass conservation between intravascular and extravascular domains leads to

$$q = \frac{d}{4} K_{\text{eff}} (C_V - C_T). \tag{n}$$

Finally, at network bifurcations, mass conservation is enforced, assuming no exchange with the tissue at the bifurcation point itself so that

$$\sum_j \phi_{ij} = 0. \tag{o}$$

where ϕ_{ij} represents the mass flux at the extremity of segment $\{ij\}$ adjacent to node i.

Equation (j) is solved using a Finite Volume approach, where each segment is divided into cylindrical subsegments of length approximately 5μm (10 per segments on average). Each subsegment then becomes a source in equation (h). We further consider the tissue domain (V_T), i.e., the subdomain within extravascular space where metabolic activity occurs, has dimensions corresponding to twice the network dimensions, to mitigate the infinite domain effects. Beyond these limits, we assume free diffusion. Typical networks used in this work had dimensions $600 \times 600 \times 50\mu\text{m}^3$. This domain is subsequently discretised into regular hexahedral elements of length approximately 25μm (10,000 elements total) and each hexahedral element becomes a sink in equation (h). We then estimate the discretized integrals derived from equation (h) following (*71*). Similar to equation (a) to (f), equations (g) to (o) form a non-linear problem that we solve iteratively.

Boundary conditions. Typical retina networks skeletons used for simulations are displayed in Figure 1B and belong to the lower retina plexus. Such networks are relatively small, square-shaped, typically including only a few hundred microvessels. As a consequence, most boundary nodes lie on the peripheral region of the network (purple dots in fig. S4F), although a number can also be found in the central region (green dots in fig. S4F). The latter correspond to vessel diving from the upper plexi.

To mitigate effects of scale while limiting computational complexity, we considered that all nodes on the peripheral region were connected to a low pressure (collector point) point and all nodes in the central region were connected to a high pressure (injector) point corresponding to the retina vein and artery respectively with a pressure drop between the two points encountered in the microcirculation (Table S3). The flowrate at these nodes is then described by

$$Q_{BC} = G_{BC}(P_i - P_{BC}), \tag{p}$$

where P_{BC} is the pressure associated with the injector/collector and G_{BC} the effective conductance defined as

$$G_{BC} = \frac{\pi d_{BC}^4}{128 \mu_{BC}(H_{d,BC}, d_{BC}) L_{BC}}, \tag{q}$$

where d_{BC} is the diameter of the unique segment neighbouring the boundary node. $H_{d,BC}$ is then taken as either the discharge haematocrit associated with such a segment or with the systemic haematocrit (Table S3) depending on whether the boundary node is an outlet or an inlet. Here, all nodes connected to the injector point are inlets (green dots in fig. S4F) and all nodes connected to the collector point are outlets (purple dots in fig. S4F). Finally, L_{BC} is the distance between each boundary node and the injector/collector points. Such a distance is estimated assuming that the networks we consider are located at mid-distance between the center and the outer rim of the retina (Table S3). As a consequence,

L_{BC} depends on the injector/collector location (fig. S4F), which is further amplified by the lack of spatial orientation of the microvascular network. To avoid favoring a specific direction, we repeat the simulations moving the injector/collector points around the network and average the results for each network.

For oxygen transport, we assume that inlet nodes have a fixed, high oxygen concentration corresponding to the saturated blood entering the microcirculation ($C_{V,BC} = \alpha_V P_{O_2,BC}$, Table S3). For outlet nodes, similar to (*76*), we assume pure convection ($D_V \nabla C_V = 0$). With regards to tissue, far-field homogeneous Dirichlet boundary condition ($C_T = 0$) is naturally enforced by the Green's function formulation.

In total, 96 simulations were run with 4 directions for the injector/collector per network ($-\boldsymbol{x}, \boldsymbol{x}, -\boldsymbol{y}, \boldsymbol{y}$), 6 networks per group, 4 groups.

Section B. Parameter values.

Model parameters were prescribed using data from the literature and are reported in table S3, We point out that the parameters are reported using units and definitions that are most common in the literature, e.g. see (*69-71*), to facilitate dissemination.

Section C. Validation.

We validated the model by solving blood flow and oxygen transport problems, i.e., equations (a) to (q), for each network of the healthy control group (repeated four times to account for injector/collector positions) and compared the prediction to *in vivo* measures found in the literature, using confocal microscopy for blood flow (*86*) and Two-photon phosphorescence lifetime microscopy for intravascular oxygen levels (*87*). Fig. S4F shows the measured (blue) and predicted (red) blood flow rates and intravascular oxygen concentration (expressed as partial pressure). Predicted values correspond to the average over all vessels, networks and repeats, and error bars are the associated standard deviation. We see that we have a good agreement between experiments and simulations, with the discrepancies primarily due to the oversimplification made by the model, the small scale of the network and the choice of boundary conditions made. Still, we argue that such limitations impact each group equally, so that relative comparison between each group remains consistent.

Section D. Integral quantities.

Integral quantities presented in the main manuscript were calculated for each network (including repeats) of each group. Such quantities are total blood flow perfusion, oxygen extraction coefficient and hypoxia susceptibility. We define the total blood flow perfusion as

$$TBF = \frac{1}{2} \sum_{i \in \{BC\}} |Q_{ij}|, \qquad \text{(r)}$$

where $\{BC\}$ is the set of all boundary nodes. We then define the extraction coefficient, i.e., the mass fraction of oxygen crossing the blood vessel walls to be consumed by the tissue as

$$E = \frac{\sum_{i \in \{BC_{in}\}} \phi_{ij} - \sum_{i \in \{BC_{out}\}} \phi_{ij}}{\sum_{i \in \{BC_{in}\}} \phi_{ij}}, \qquad \text{(s)}$$

where $\{BC_{in}\}$ and $\{BC_{out}\}$ are the sets of inlet and outlet boundary nodes connected to the high-pressure injector and low-pressure collector points.

Finally, we define the elementary hypoxia susceptibility coefficient as

$$hy_i = \frac{\iiint_{V_T} H(C_T < C_{\tau.i})dV}{\iiint_{V_T} H(C_T)dV}, \quad \text{(t)}$$

where H is the Heaviside function and $C_{\tau.i}$ the hypoxia threshold. To avoid choosing a specific value for such a threshold we evaluate the elementary hypoxia susceptibility coefficient for multiple threshold values, i.e., $C_{\tau,i} \in \{0 \ldots C_{V,BC}\}$ where $C_{V,BC} = \alpha_V P_{O_2,BC}$ and obtain a distribution of elementary hypoxia susceptibility coefficient instead. We then consider the median of such a distribution so as not to be attached to a specific threshold and can be considered a footprint of the network ability to deliver oxygen to tissue.

Primers and probes. Mouse *Lrg1* (FOR-CCAATAACTCTCTGTCCAGCACG; REV-TCTTGTTTCGGTTGGCGACCAG), mouse *Vegfa* (FOR-TCTGGAAGTGAGCCAATGTG; REV-GACTTGTGTTGGGAGGAGGA), mouse *Tgfβ* (FOR-TTGCTTCAGCTCCACAGAGA; REV-TGGTTGTAGAGGGCAAGGAC), mouse *Hif1*α (FOR-TGAGCTTGCTCATCAGTTGC; REV-CATAACAGAAGCTTTATCAAGATGTGA) , mouse *Gapdh* (FOR-ACTGAGGACCAGGTTGTCTCC; REV-CTGTAGCCGTATTCATTGTCATACC), human *LRG1* (FOR- TGCTGGATCTAACCCGAAAC; REV- AGAGCTTTCAGGCCGTGTAG), human *SNAIL* (FOR-TCGGAAGCCTAACTACAGCGA; REV-AGATGAGCATTGGCAGCGAG), human *ACTA2* (FOR-CCGACCGAATGCAGAAGGAG; REV-ACAGAGTATTTGCGCTCCGAA), human *FN1* (FOR-GTGTGATCCCGTCGACCAAT; REV-CGACAGGACCACTTGAGCTT), human *MMP2* (FOR-GAGTGCATGAACCAACCAGC; REV-GTGTTCAGGTATTGCATGTGCT), human *COL1A2* (FOR-TGCTTGCSGTAACCTTATGCCTA; REV-CAGCAAAGTTCCCACCGAGA), human *VCAM1* (FOR-GATTCTGTGCCCACAGTAAGGC; REV-TGGTCACAGAGCCACCTTCTTG), human *GAPDH* (FOR-AAGGTGAAGGTCGGAGTCAA; REV-AATGAAGGGGTCATTGATGG), Mouse *Lrg1* probes (RNAscope, Mm-Lrg1 cat no. 423381).

Real time PCR. Total RNA was extracted from tissues or cells using the RNeasy Mini Kit (Qiagen) according to the manufacturer's protocol. RNA concentration and purity were determined using a NanoDrop spectrophotometer (Thermo Fisher Scientific). Total RNA (1 μg) was reverse transcribed into cDNA using the QuantiTec Reverse Transcription Kit (#205311, Qiagen) according to the manufacturer's instructions. Real-time PCR was performed using the PowerUp SYBR Green Master

Mix (Thermo Fisher Scientific) and gene-specific primers. The geometric means of the housekeeping genes GAPDH, beta actin and 18S were used as endogenous controls to normalize the expression of the target genes. Reactions were performed on a QuantStudio 6 Flex System (Thermo Fisher Scientific) with the following cycling conditions: 50°C for 2 min, 95°C for 10 min, followed by 40 cycles of 95°C for 15 sec and 60°C for 1 min, 95°C for 15 sec, 60°C for 1 min, 95°C for 15 sec. The relative expression of the target genes was calculated using the comparative CT (ΔΔCT) method.

Supplementary Figures.

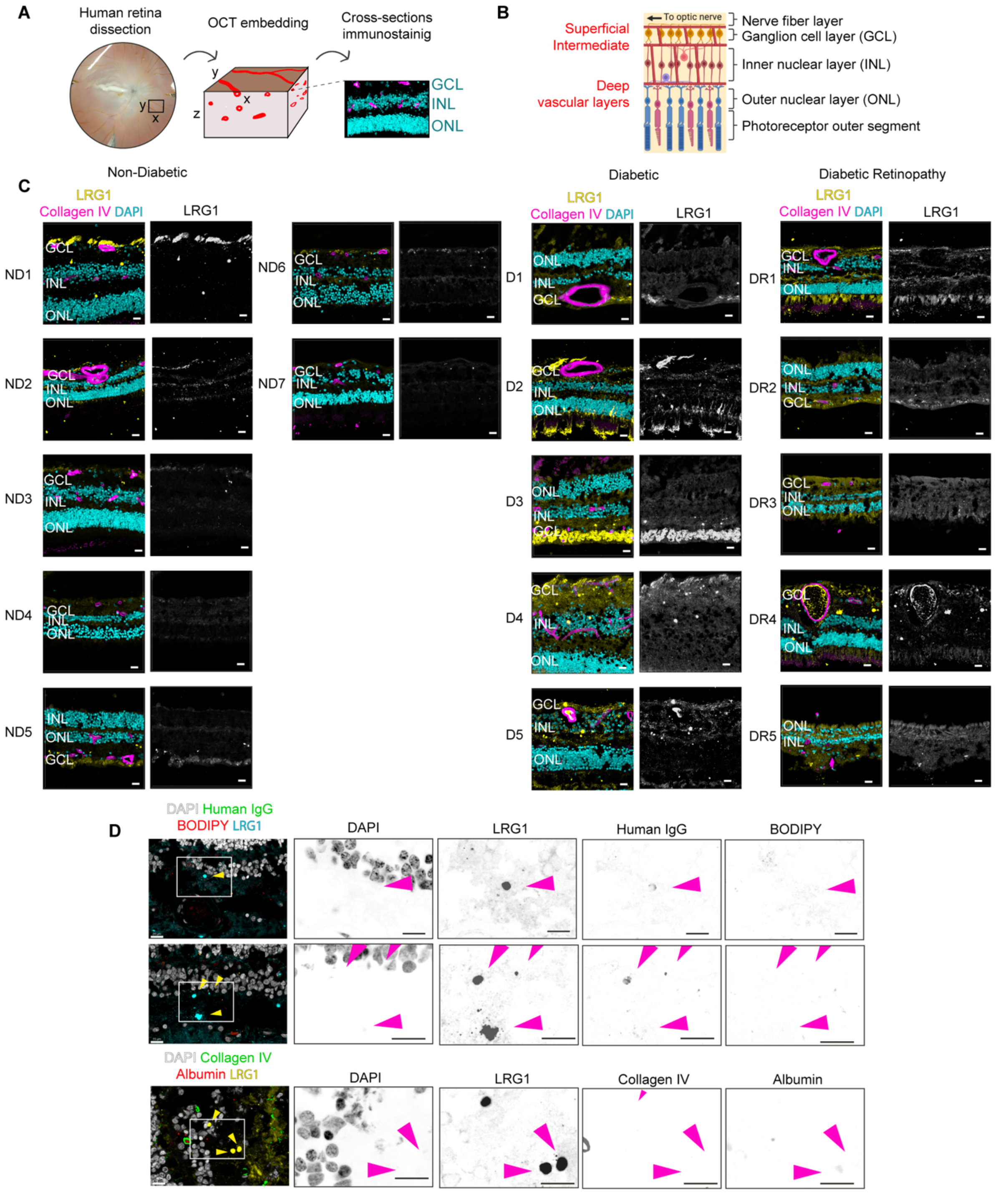

Figure S1. LRG1 expression in human retina.

(**A**) Diagram showing the orientation of the cryosections in respect to the retinal tissue. (**B**) Diagram showing the position of the three retinal vascular layers within the inner retina. Created with BioRender.com. (**C**) Confocal images of post-mortem human retinal sections immuno-stained for LRG1 (yellow), blood vessels (Collagen IV, magenta) and nuclei (DAPI, cyan). The ganglion cell layer

(GCL), inner nuclear layer (INL) and outer nuclear layer (ONL) are marked. One representative per group (ND7, D2 and DR4) are reused in Fig. 1A. Scale bar, 20 µm. (**D**) Top two panels, sections from DR2 immunostained for nuclei (DAPI, white), lipid droplets (BODIPY, red), LRG1 (cyan). Bottom panel, sections from DR5 immunostained for nuclei (DAPI, white), blood vessels (collagen IV, green), albumin (red), LRG1 (yellow). Magenta arrows in the high magnification single channels indicate areas of clustered LRG1 signal. Scale bar, 15 µm.

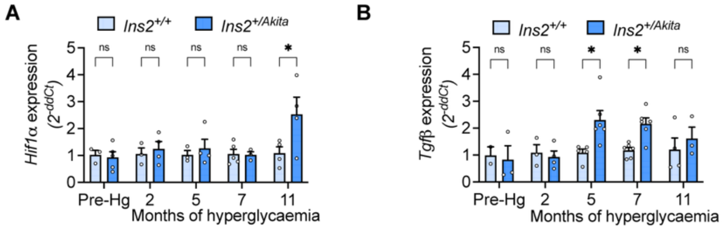

Figure S2. Retinal expression of *Hif1α* and *Tgfβ* following hyperglycemia. (**A**) *Hif1α* and (**B**) *Tgfβ* expression in retinas isolated from control (*Ins2*$^{+/+}$) and diabetic (*Ins2*$^{+/Akita}$) mice at different time-points following hyperglycaemia or one-month prior to the onset (Pre-Hg). n=4-7 mice per group. 2-way Anova; Šidák's test for multiple comparisons. All data points represent mean and standard error of the mean. $P^* < 0.05$.

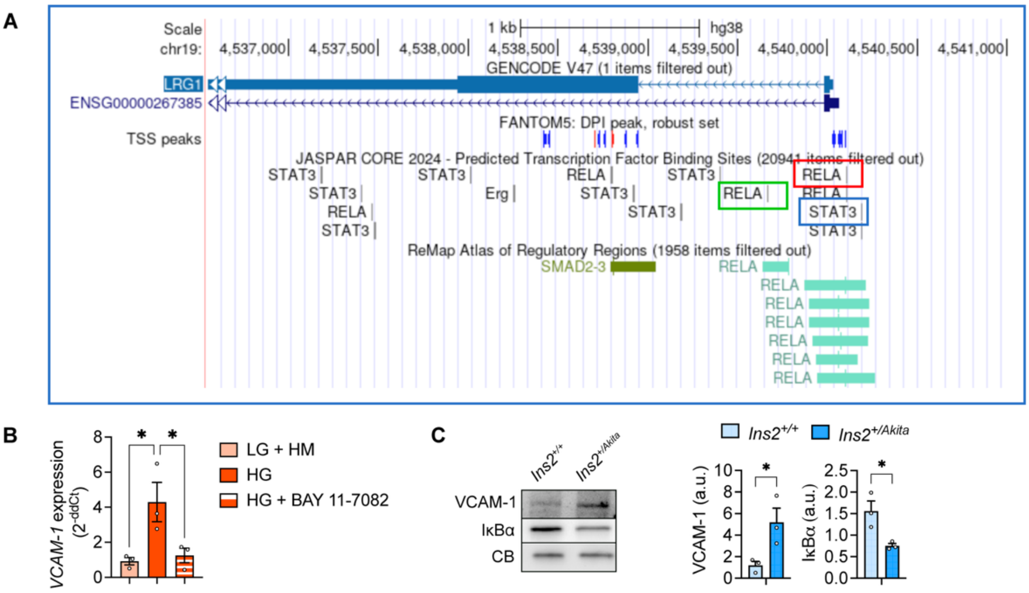

Figure S3. LRG1 expression is mediated by the NF-κB pathway.

(A) Analysis of Human (GRCh38/hg38) Chromosome 19 (p13.3) via UCSC Genome Browser to identify STAT3 (blue box) and two RELA (RELA 1 red box, RELA 2 green box) transcription factor binding sites within the LRG1 promoter region, proximal to the transcription start site (TSS, FANTOM5). Binding sites were identified using both predictive algorithms (JASPAR CORE 2024) and empirical ChIP-Seq data (ReMap). (**B**) *VCAM1* expression in HRECs treated for 48 hours with low glucose (LG + HM), or high glucose (HG) alone or with 1 µM NF-κB inhibitor BAY 11-7082. n=3 independent experiments. 1-way Anova; Šidák's test for multiple comparisons. (**C**) VCAM-1 and IκBα expression in retinal lysates from control (*Ins2*$^{+/+}$) and hyperglycaemic (*Ins2*$^{Akita/+}$) mice following 2 months of hyperglycaemia. Blots are representative of n=3 mice per group. Arbitrary units (a.u.) represent the relative expression of the indicated proteins normalised to the loading control, Cyclophilin B (CB). Unpaired two-tailed t test.

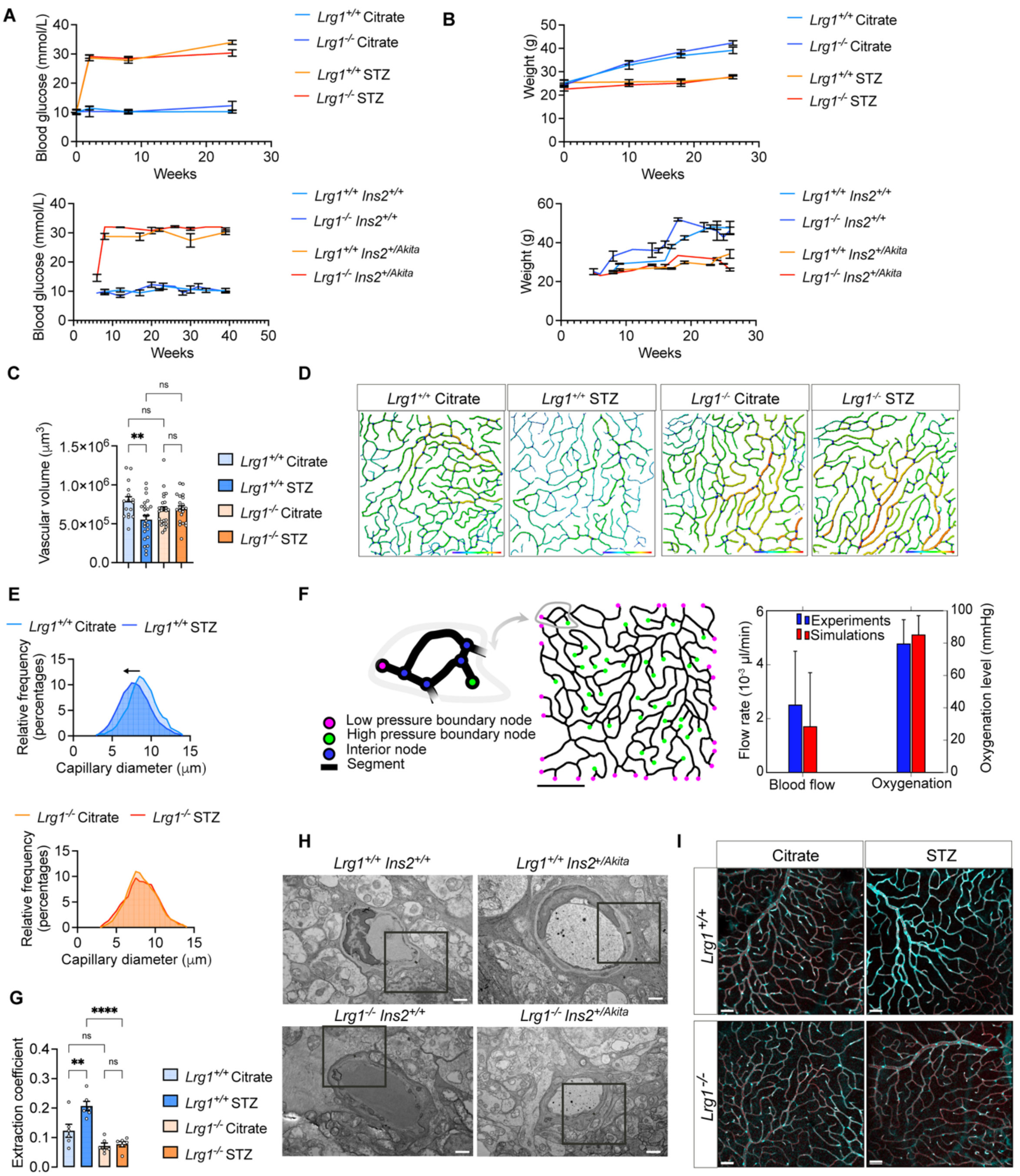

Figure S4. LRG1 promotes vascular dysfunction in the hyperglycemic retina.

(**A**) Glucose levels measured through tail prick and (**B**) weight of mice at the indicated age. n=3-9 per time point. (**C**) Vascular volume within the field of view (580x580x26 μm^3) in the retinal deep-plexus of STZ diabetic mice at 6 months post-hyperglycaemia or age-matched controls. n=7-9 mice per group. 1-way Anova; Šidák's test for multiple comparisons. (**D**) Segmentation of representative images in Fig. 2C. Capillaries between 2 branching points are color-coded based on their average diameter. (**E**) Relative frequencies of capillary diameters. Arrow indicates the shift towards narrower diameters in

wild-type diabetic mice compared to controls (*Lrg1*$^{+/+}$ citrate vs *Lrg1*$^{+/+}$ STZ, mean 8.86 Std. deviation 1.87 vs 7.96 1.93). (**F**) On the left, network approach adopted for the blood flow simulation. Green nodes connected to the high-pressure injector point and purple nodes connected to the low-pressure collector point. In the middle, typical retinal microvascular network used for simulation (taken from the healthy control group), with boundary nodes highlighted and the subset presented in left panel highlighted in grey. Scale bar, 150 μm. On the right, comparison between experiment (blue) and simulation (red) blood flow rate (left) and intravascular oxygen levels (right), with standard deviation. Experimental values taken from (85) for blood flow and (86) for oxygen levels. (G) Quantification of hypoxia index based on segmented images. n=6 mice per group. 1-way Anova; Tukey's test for multiple comparisons. (**H**) Original TEM images of retinas from control (*Ins2*$^{+/+}$) and diabetic (*Ins2*$^{+/Akita}$) mice of either *Lrg1*$^{+/+}$ or *Lrg1*$^{-/-}$ background after 6 months of hyperglycaemia shown in Fig. 2H (region in squares). Scale bar, 1 μm. (**I**) Confocal images of retinal deep vasculature of STZ mice following 6 months of hyperglycaemia, Collagen IV (cyan), PECAM-1 (red). Scale bar, 50 μm. All data points represent mean and standard error of the mean. $P^{**}<0.01$; $P^{****}<0.0001$.

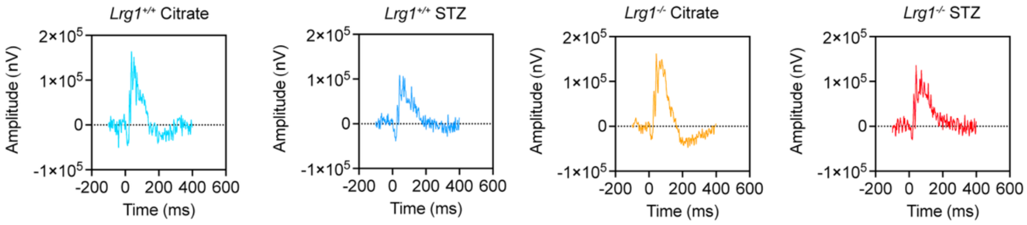

Figure S5. ERG in STZ diabetic mice.

Representative scotopic ERG waveforms recorded after short dark adaptation (flash stimulus, intensity 50 cd·s/m²) in the 4 experimental groups. Traces show representative ERG response amplitude (nV) over time (ms). Recordings were performed after 6 months of hyperglycaemia. n=6-9 mice per group.

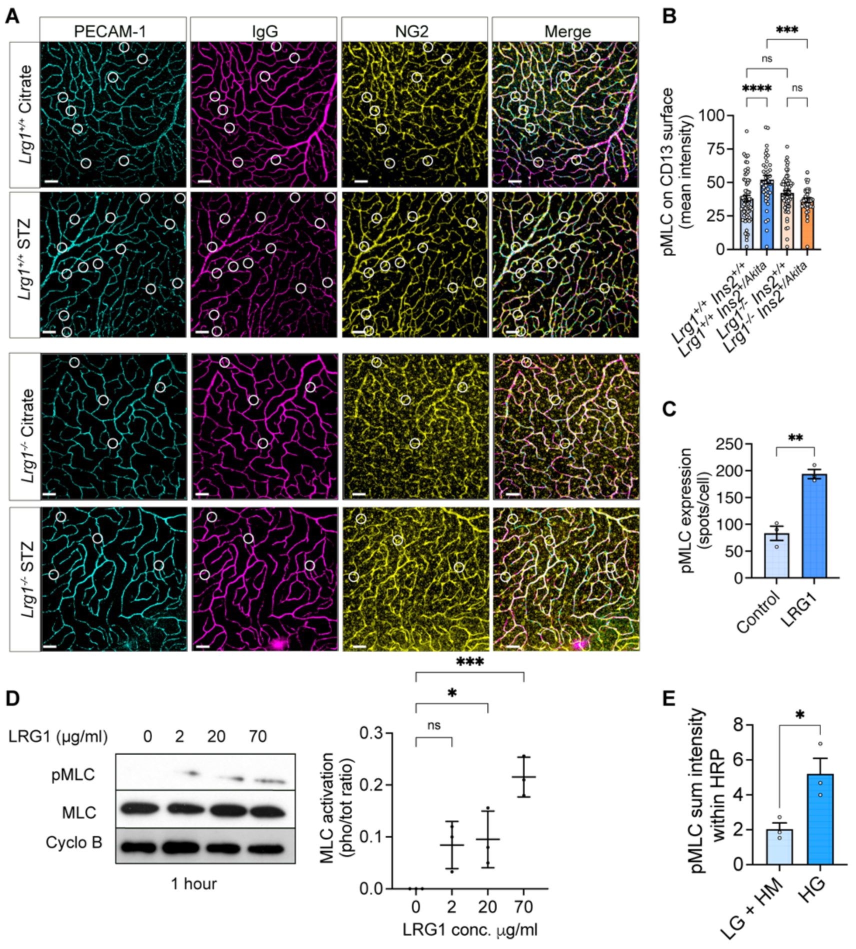

Figure S6. LRG1 promotes pericyte dysfunction.

(**A**) Representative confocal images of the retinal deep vascular plexus at 6 months post-hyperglycaemia onset in the STZ diabetic mice and their controls. White circles indicate PBs. n=6-19 mice per group, cumulative of 3 independent experiments. (**B**) Quantification of pMLC expression within pericytes (CD13-based mask). n=40-70 single pericytes, 4 images/group. 1-way Anova; Šidák's test for multiple comparisons. (**C**) Quantification of pMLC^{+} spots within human retinal pericytes (HRPs) treated with 70 µg/ml of recombinant human LRG1. n=3 independent experiments. Unpaired two-tailed t test. (**D**) HRPs treated for 1 hour with increasing concentration of LRG1 and probed for pMLC, total MLC and Cyclo B (loading control). MLC activation expressed as ratio of normalised phosphorylated MLC (pMLC) to normalised total MLC. n=3 independent experiments. (**E**) Quantification of pMLC expression within pericytes (cmfda-based mask). n=3 independent experiments, 4 images/group. Unpaired two-tailed t test. $P^{**}<0.01$; $P^{***}<0.001$; $P^{****}<0.0001$.

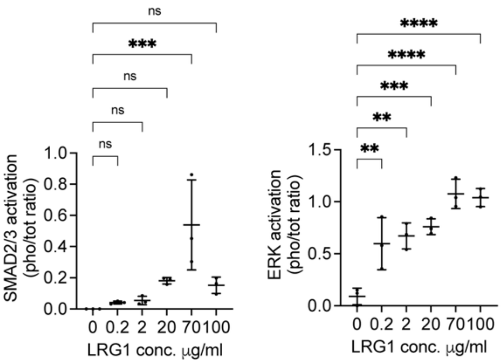

Figure S7. LRG1 activates canonical and non-canonical TGFβ signaling in HRPs.

HRPs treated for 1 hour with increasing concentrations of LRG1 and probed for pSMAD2/3, total SMAD2/3, pERK, total ERK and Cyclo B (loading control). SMAD2/3 (left) and ERK (right) activations expressed as ratio of normalised phosphorylated to normalised total (representative blots in 3F). All data points represent mean and standard deviation of 3 independent experiments. $P^{*}<0.05$; $P^{**}<0.01$; $P^{****}<0.0001$.

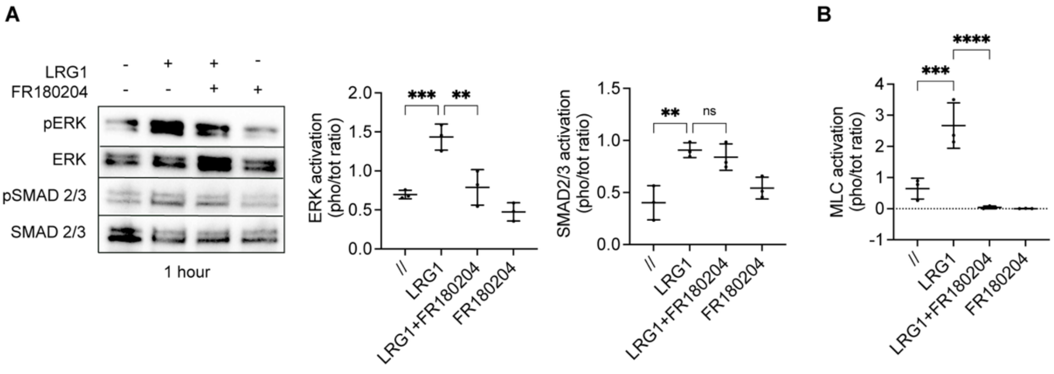

Figure S8. LRG1 promotes MLC activation through ERK.

(**A**) HRPs treated for 1 hour with 70 µg/ml of LRG1 in the presence or absence of the ERK inhibitor FR180204 at 50 µM and probed for pERK, total ERK, pSMAD2/3, total SMAD2/3. ERK (left) and SMAD2/3 (right) activations expressed as ratio of normalised phosphorylated to normalised total. "\\" indicates untreated. (**B**) MLC activation expressed as ratio of normalised phosphorylated to normalised total (representative blot in fig. 3G). "\\" indicates untreated. All data points represent mean and standard deviation of 3 independent experiments. $P^{*}<0.05$; $P^{**}<0.01$; $P^{***}<0.001$.

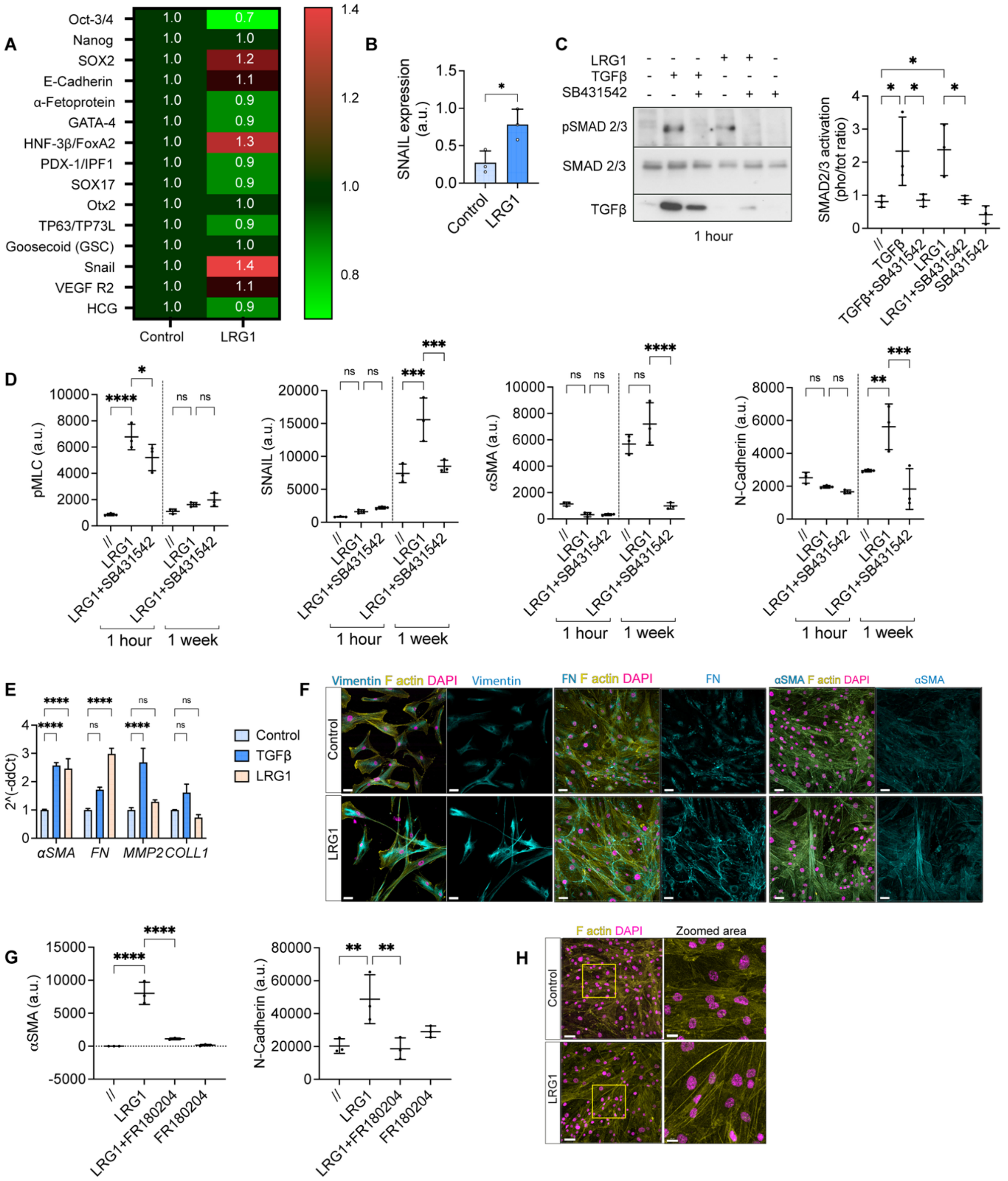

Figure S9. LRG1 promotes pericyte transition to contracted/fibrotic state.

(**A**) Cell lysates from HRPs treated with 70 µg/ml LRG1 for 1 week analysed with semi-quantitative antibody array. n=1. (**B**) *SNAIL* expression in HRPs treated with 70 µg/ml LRG1 for 1 week. n=3 independent experiments. Unpaired two-tailed t test. (**C**) HRPs treated for 1 hour in complete medium with 70 µg/ml of LRG1 in the presence or absence of ALK5 inhibitor SB431542 at 10 µM and TGFβ at 5 ng/ml and probed for pSMAD2/3, total SMAD2/3 and Cyclo B. SMAD2/3 activation expressed as

ratio of normalised phosphorylated to normalised total. n=3 independent experiments. "\\" indicates untreated. (**D**) Quantifications of western blot in Fig. 3I. Arbitrary units (a.u.) represent the relative expression of the protein indicated normalised to the loading control (Cyclo B). n=3 independent experiments. "\\" indicates untreated. (**E**) *αSMA*, fibronectin (*FN*), matrix metalloproteinase 2 (*MMP2*) and Collagen type-1 (*COLL1*) gene expression in HRPs treated with 70 μg/ml LRG1 for 1 week. n=3. 2-way Anova; Tukey's test for multiple comparisons. (**F**) Confocal images of HRPs treated with 70 μg/ml LRG1 for 1 week expressing Vimentin, FN and αSMA (all in cyan). Scale bar, 50 μm. Images are representative of 3 independent experiments. (**G**) Quantifications of western blot in Fig. 3J. Arbitrary units (a.u.) represent the relative expression of the protein indicated normalised to the loading control, (Cyclo B). n=3 independent experiments. "\\" indicates untreated. (**E**) Confocal images of HRP treated with 70 μg/ml LRG1 for 1 week expressing F actin-rich stress fibers (yellow). Scale bar, 50 μm. Images are representative of 3 independent experiments. All data points represent mean and standard error of the mean. $P^{***}<0.001$; $P^{****}<0.0001$.

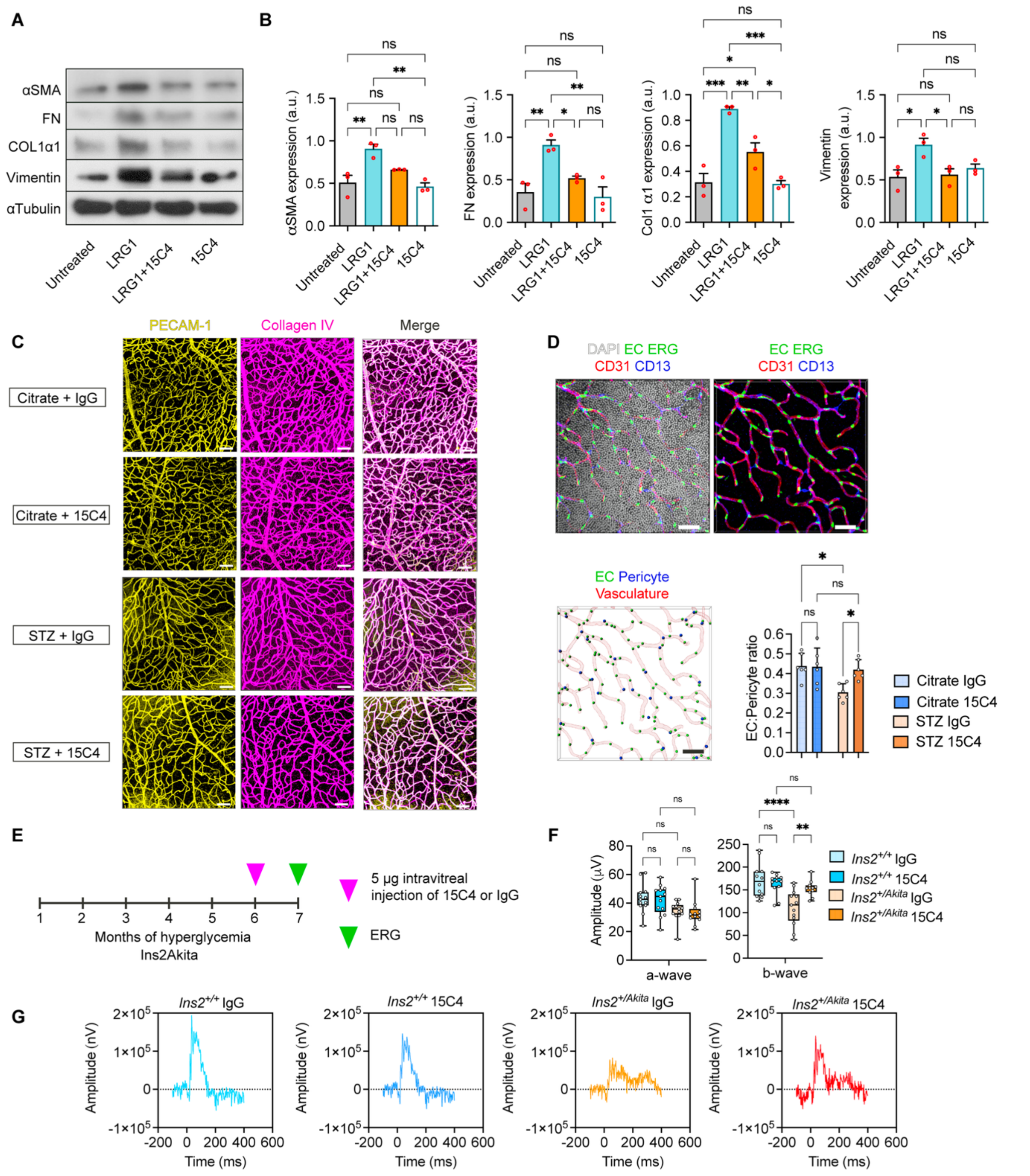

Figure S10. Blocking LRG1 prevents pericyte phenotypic switch and attenuates hyperglycemia-induced neuroretina dysfunction.

(**A**) Cell lysates of human retinal pericytes (HRP) treated with 70 µg/ml recombinant human LRG1 (LRG1) and 500 µg/ml µM of 15C4 for 1 week and blotted for α smooth muscle actin (αSMA), fibronectin (FN), Collagen 1α1 (COL1α1), Vimentin and αTubulin as loading control. (**B**) Semi-quantification of 3 independent experiments. Arbitrary units (a.u.) represent the relative expression of

the protein indicated normalised to the loading control, Cyclophilin B (Cyclo B). 1-way Anova; Tukey's test for multiple comparisons. (**C**) Single channel confocal images of the retinal vasculatures shown in Fig. 4H. n=6 mice per group. (**D**) Quantification of the EC to pericyte ratio across the 4 experimental groups. ECs identified as ERG^+ nuclei (green), and pericytes as $DAPI^+$ nuclei colocalising with the pericyte marker CD13 (blue). 3D Imaris rendering illustrates the spatial distribution of ECs (green dots) and pericytes (blue dots) relative to the vasculature (red surface). n = 6 mice per group. (**E**) Ins2Akita mice with 6 months of hyperglycaemia and controls were injected with 5 μg of 15C4 in one eye and 5 μg of IgG1 control in the contralateral eye. (**F**) A- and b-wave ERG response after short dark adaptation recorded 1 month after injections (7 months of hyperglycaemia in total). n=6-9 mice per group. 1-way Anova; Tukey's test for multiple comparisons. (**G**) Representative scotopic ERG waveforms recorded after short dark adaptation (flash stimulus, intensity 50 cd·s/m²) in the four experimental groups. Traces show representative ERG response amplitude (nV) over time (ms). $P^{*}<0.05$; $P^{**}<0.01$; $P^{***}<0.001$; $P^{****}<0.0001$.

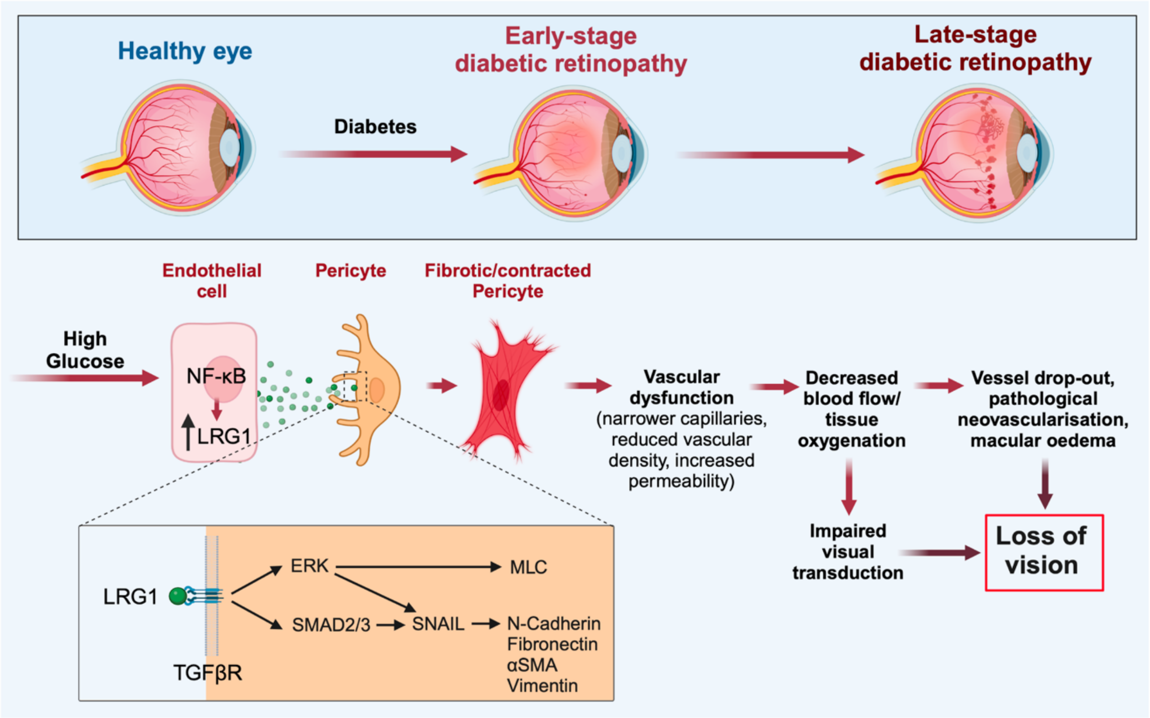

Figure S11. Proposed mode of action of LRG1 in DR.

In the early stage of diabetic retinopathy, chronic high glucose concentrations activate the expression and secretion of LRG1 by retinal endothelial cells in a NF-κB-dependent manner. Increased LRG1 in turns activate TGFβ canonical (SMAD2/3-mediated) and non-canonical (ERK-mediated) signalling in pericytes, driving upregulation of cytoskeletal and extracellular matrix proteins promoting their transition to a more fibrotic and contracted phenotype. This sustained contraction of pericytes leads to the narrowing of capillary lumens and thickening of their basement membrane with adverse effects on retinal blood flow and oxygen delivery to retinal neurons, ultimately impacting visual transduction. Moreover, the exacerbation of vessel dropout and increased permeability can eventually trigger the growth of new vessels (PDR) and the leakage of fluids into the macula (DME), leading to severe vision impairment and blindness. Created with BioRender.com.

Table S1. Demographics and clinical details of human retinal donors.

NSCLC (Non-small cell lung carcinoma), TIA (Transient ischaemic attack), T2DM (Type 2 diabetes mellitus), PVD (Peripheral vascular disease), AKI (Acute kidney injury), (COPD) chronic obstructive pulmonary disease, MI (myocardial infarction). Retinal LRG1 expression measured on immunostained cross-sections (fig. S1A) is expressed as mean fluorescence intensity (MFI).

ID	Type	Gender	Age	Ocular Disease	Systemic Diseases	Cause of Death	Retinal LRG1 expression (MFI)
ND1	Non Diabetic	F	44	None	NSCLC	Metastatic NSCLC	1.87
ND2	Non Diabetic	F	59	None	None	Spontaneous Subarachnoid Haemorrhage	4.84
ND3	Non Diabetic	F	62	None	Osteoarthritis, TIA	Spontaneous Intracerebral Haemorrhage	0.71
ND4	Non Diabetic	F	73	Cataracts	NSCLC	Metastatic NSCLC	7.72
ND5	Non Diabetic	M	50	None	Liver Cirrhosis	Liver Cirrhosis with Multi Organ Failure	7.38
ND6	Non Diabetic	M	51	None	Liver Cirrhosis	Liver Cirrhosis with Portal Hypertension	1.90
ND7	Non Diabetic	M	63	None	NSCLC	Metastatic NSCLC	2.71
D1	Diabetic	M	65	None	T2DM	Infected Diabetic Fat, PVD, Hypercalcaemia, Non Traumatic AKI	10.70
D2	Diabetic	M	69	Myopia	DM, COPD, MI, Arthritis	Multi Organ Failure, Sepsis of unknown source	47.22
D3	Diabetic	M	70	None	DM	Multi Organ Failure, Sepsis of unknown origin, Congestive Cardiac Failure	16.52
D4	Diabetic	M	77	None	DM	Metastatic Prostate	11.31

						Cancer, Renal Cell Cancer	
D5	Diabetic	M	82	None	DM	Heart Failure, Myocardial Infarction	36.50
DR1	Diabetic Retinopathy	F	45	DR and DME	T2DM, MI, Asthma	Cardiogenic Schock, Ischaemic Cardiomyopathy	26.05
DR2	Diabetic Retinopathy	F	64	DR	T2DM, Neuropahty	Hypoxic Brain Injury, Cardiac Arrest, Diabetic Ketoacidosis	17.50
DR3	Diabetic Retinopathy	M	43	DR	DM	Coronary Artery Thrombus	26.95
DR4	Diabetic Retinopathy	M	51	DR	DM, COPD, Schizophrenia, Heart Failure, Psoriasis	Unknown	14.96
DR5	Diabetic Retinopathy	M	65	DR	DM	Cardiac Arrest, Cardiac Ischaemia	24.02

Table S2. Antibodies. Antibodies used in this study

Target	Company	Product	Clone	Application	Dilution
**Mouse LRG1**	Santa Cruz	Sc-390920	C-4	WB	1:1000
**Human LRG1**	Proteintech	13224-1-		WB	1:1000
**pSMAD2/3**	Cell Signaling	Mab 8828	D27F4	WB	1:500
**SMAD2/3**	Cell Signaling	Mab 8685	D7G7	WB	1:1000
**pERK1/2**	Cell Signaling	9102		WB	1:1000
**ERK1/2**	Cell Signaling	4695		WB	1:1000
**Cyclophilin B**	R&D Systems	Mab5410		WB	1:1000
**TGFβ**	Cell Signaling	Mab 1835	56E4	WB	1:1000
**pMLC**	Cell Signaling	3671		WB	1:1000
**MLC**	Cell Signaling	3672		WB	1:1000
**SNAIL**	Abcam	Ab180714		WB	1:1000
**αSMA**	Cell Signaling	Mab19245	D4K9N	WB	1:500
**N-Cadherin**	Cell Signaling	4061		WB	1:1000
**Fibronectin**	Merck	Ab2033		WB	1:500
**Col1α1**	Cell Signaling	Mab 91144	E8I9Z	WB	1:500
**Vimentin**	Cell Signaling	4970		WB	1:2000
**αTubulin**	Cell Signaling	2144		WB	1:2000
**VCAM-1**	Cell Signaling	13662S	E1E8X	WB	1:1000
**IκBα**	Cell Signaling	4812S		WB	1:1000
**CD13**	R&D Systems	AF2335		IF retina	1:200
**Collagen IV**	EMD Millipore	Ab769		IF retina	1:200
**PECAM-1**	BD Pharminogen	553370	MEC13.3	IF retina	1:50

**IsolectinGS-IB4-Alexa-568**	Invitrogen	I21412		IF retina	1:200
**Fibronectin**	Merck	Ab2033		IF retina	1:100
**NG2**	Millipore	Ab5320		IF retina	1:200
**ERG**	Abcam	Ab92513	EPR3864	IF retina	1:100
**Vimentin**	Abcam	Ab92547		IF cells	1:1000
**Rhodamine-Phalloidin**	Life Technologies	R415		IF cells	1:1000
**SNAIL**	EMD Millipore	Mabe167	10H4.1	IF cells	1:200
**αSMA**	Abcam	Ab5694		IF cells	1:200
**Collagen IV Alexa Fluor™ 488**	eBiosciences	53-9871-82	1042	IF (human)	1:200
**Human IgG-FITC**	Invitrogen	31535		IF (human)	1:400
**Albumin**	NovusBiologicals	NB600-		IF (human)	1:100
**Human Fc Block**	BD Biosciences	564220	Fc1	IF (human)	1:200
**Donkey anti-Rabbit IgG (H+L) Alexa Fluor™ 594**	Thermo Fisher Scientific / Invitrogen	A-21207			Dilution specified in methods
**Donkey anti-Rat IgG (H+L) Alexa Fluor™ 488**	Thermo Fisher Scientific / Invitrogen	A-21208			Dilution specified in methods

**Donkey anti-Goat IgG (H+L) Alexa Fluor™ Plus 647**	Thermo Fisher Scientific / Invitrogen	A-32849			Dilution specified in methods
**Goat anti-Mouse IgG (H+L) Alexa Fluor™ 555**	Thermo Fisher Scientific / Invitrogen	A-21422			Dilution specified in methods
**Donkey anti-Rabbit IgG (H+L) Alexa Fluor™ Plus 647**	Thermo Fisher Scientific / Invitrogen	A-32795			Dilution specified in methods
**Donkey anti-Rabbit IgG (H+L) Alexa Fluor™ Plus 488**	Thermo Fisher Scientific / Invitrogen	A-32790			Dilution specified in methods

Table S3: Blood flow and oxygen transport parameters. Parameters taken from the literature to devise the oxygen flow simulation.

**Parameter**	**Unit**	**Value**	**Ref.**
Systemic haematocrit ($H_{d,BC}$)	N/A	0.4	(*77*)
Oxygen diffusion coefficient in blood (D_V)	$10^3 \mu m^2 \cdot s^{-1}$	2.4	(*78*)
Oxygen diffusion coefficient in tissue (D_T)	$10^3 \mu m^2 \cdot s^{-1}$	2.4	(*79*)
Oxygen maximum Metabolic rate ($M_{\max}$)	$10^{-2} cm^3 O_2 \cdot cm^{-3} \cdot min^{-1}$	2.5	(*69*)
P_{O_2} at half maximal metabolic rate ($P_{1/2} = \frac{C_{1/2}}{\alpha_T}$)	mmHg	10	(*80*)
Pressure drop injector-collector (ΔP_{BC})	mmHg	50	(*81*)
Oxygen partial pressure injector ($P_{O_2,BC} = \frac{C_{V,BC}}{\alpha_V}$)	mmHg	85	(*82*)
Distance network-injector/collector (L_{BC})	µm	1000	(*83*)
Endothelium thickness (e)	µm	2	(*84*)
P_{O_2} at half maximal haemoglobin saturation ($P_{50} = \frac{C_{50}}{\alpha_b}$)	mmHg	40	(*85*)
Hill's exponent (n)	N/A	2.5	(*85*)
Oxygen binding capacity of red blood cells (C_0)	$cm^3 O_2 \cdot cm^{-3}$	0.5	(*79*)
Effective solubility of oxygen in blood (α_V)	$10^{-5} cm^3 O_2 \cdot cm^{-3} \cdot mmHg^{-1}$	3.1	(*71*)
Effective solubility of blood in tissue (α_T)	$10^{-5} cm^3 O_2 \cdot cm^{-3} \cdot mmHg^{-1}$	3.9	(*79*)

Original Western Blots

Fig. 1C

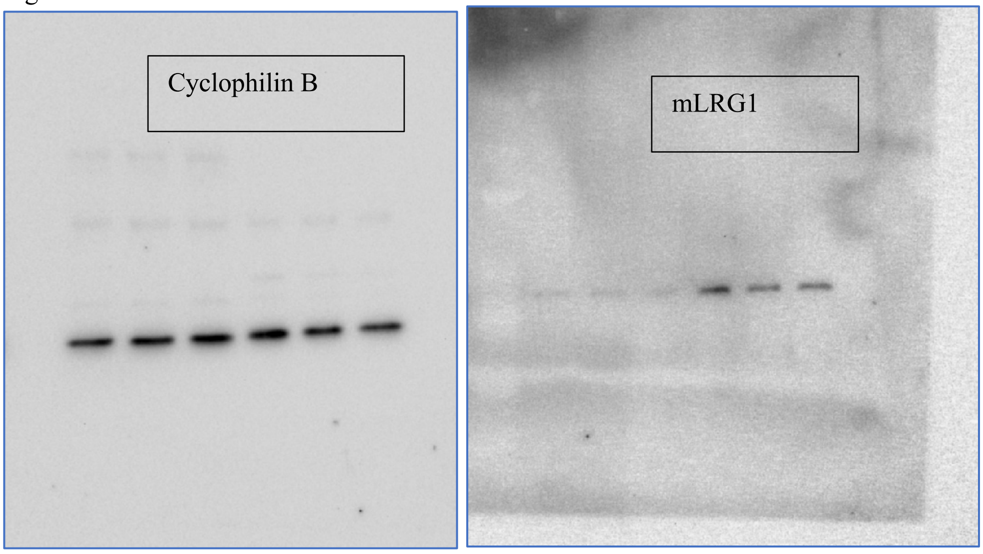

Fig. 1I

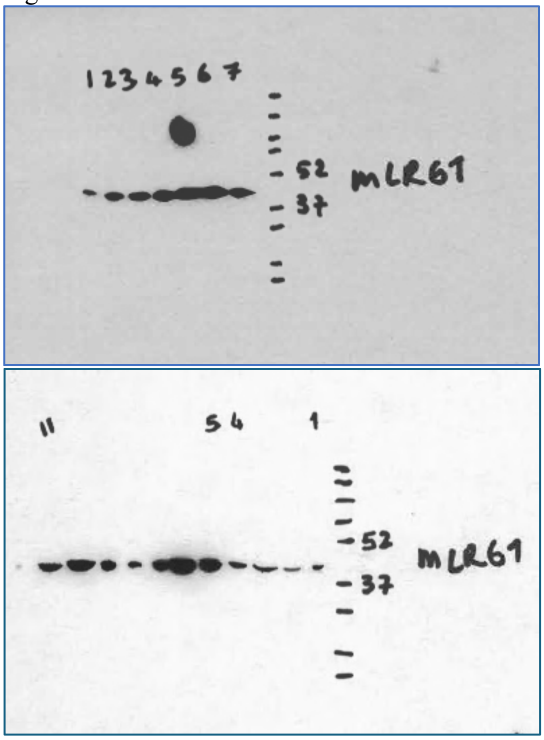

Fig. 3F

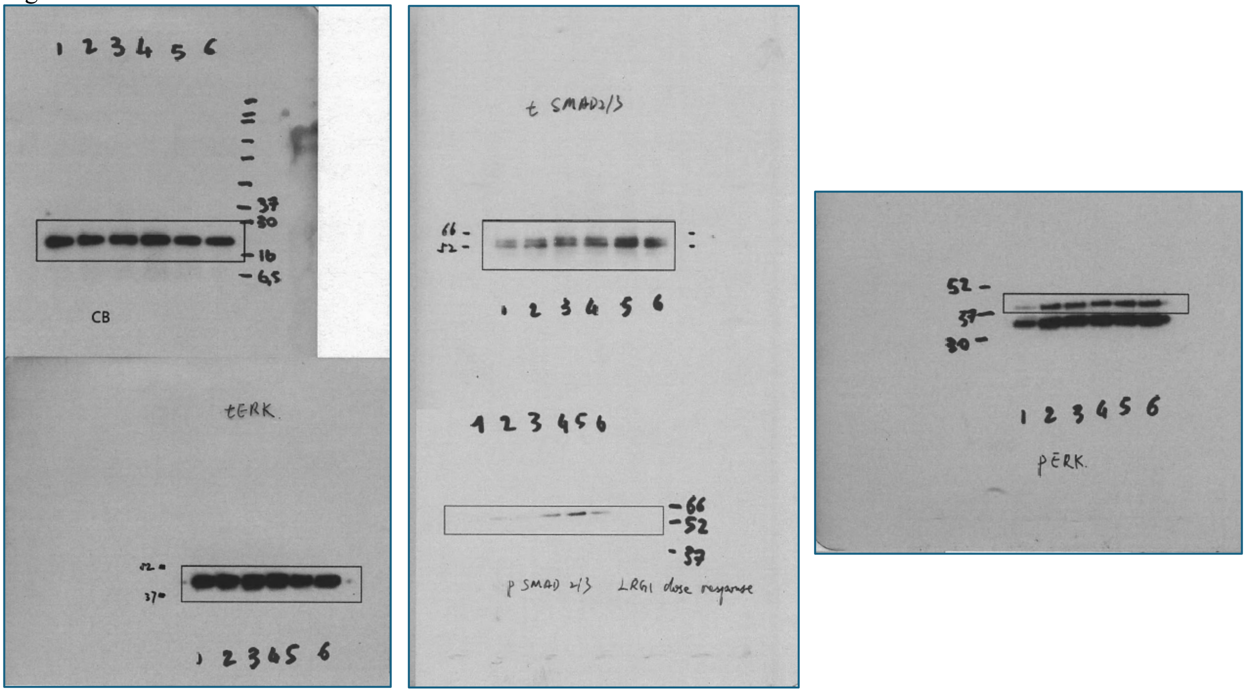

Fig. 3G

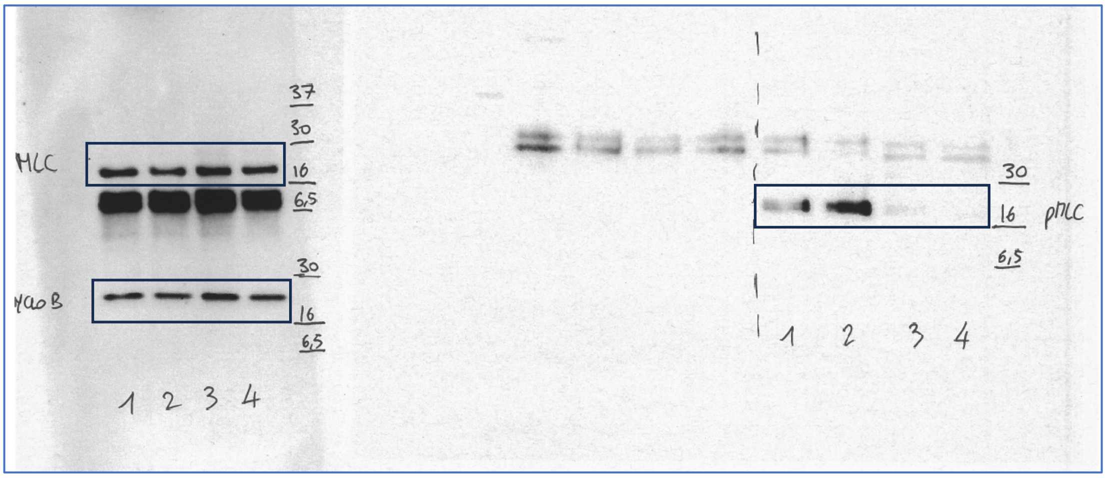

Fig. 3H

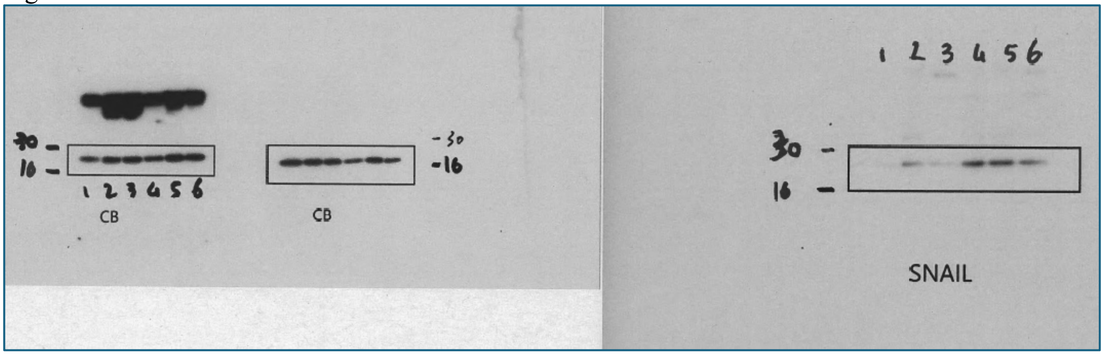

Fig. 3I

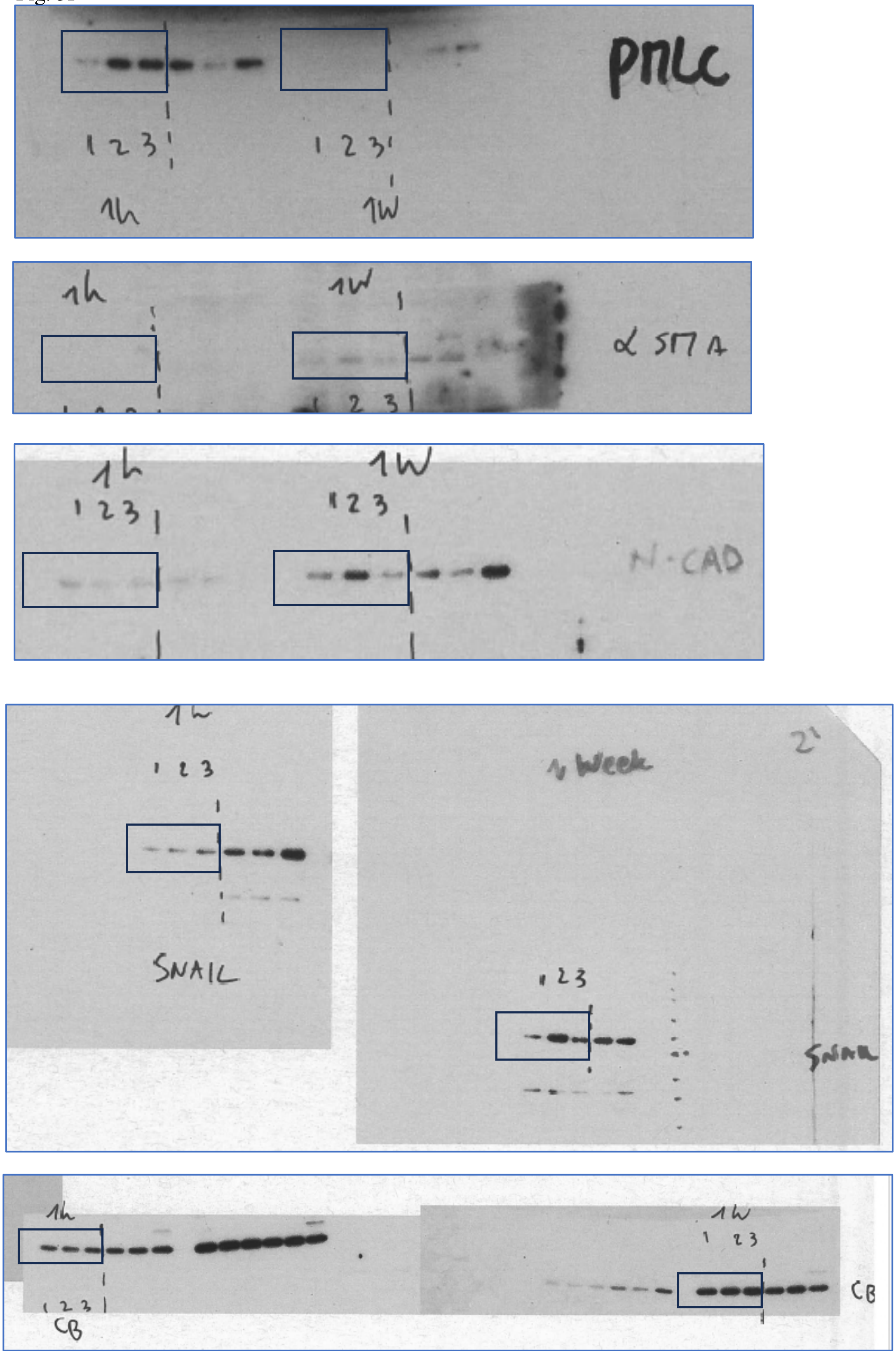

Fig. 3J

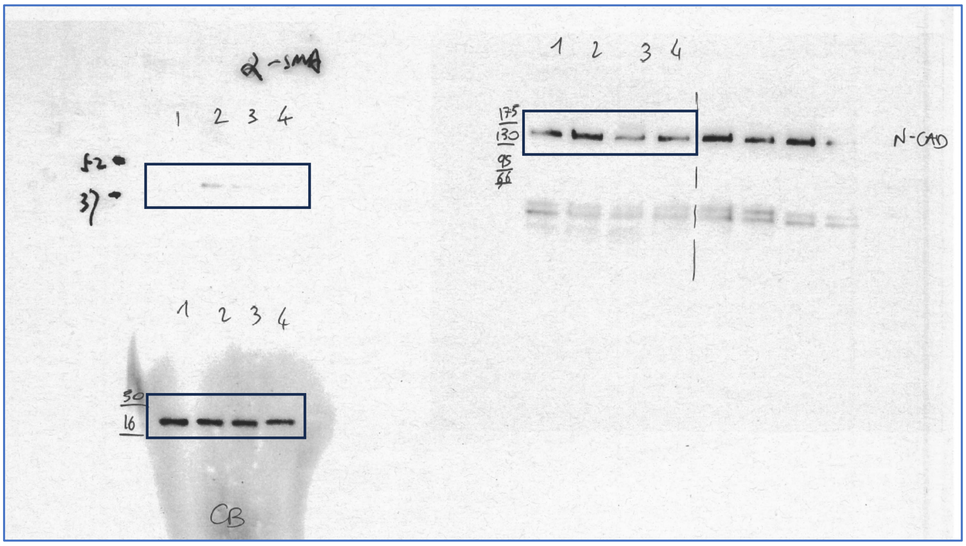

Fig. S3C

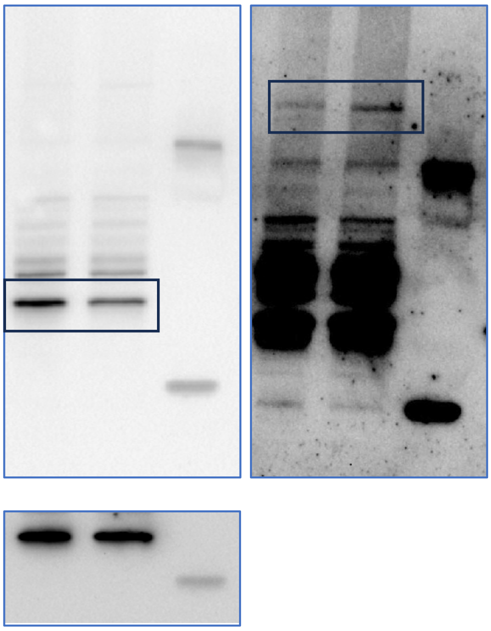

Fig. S6D

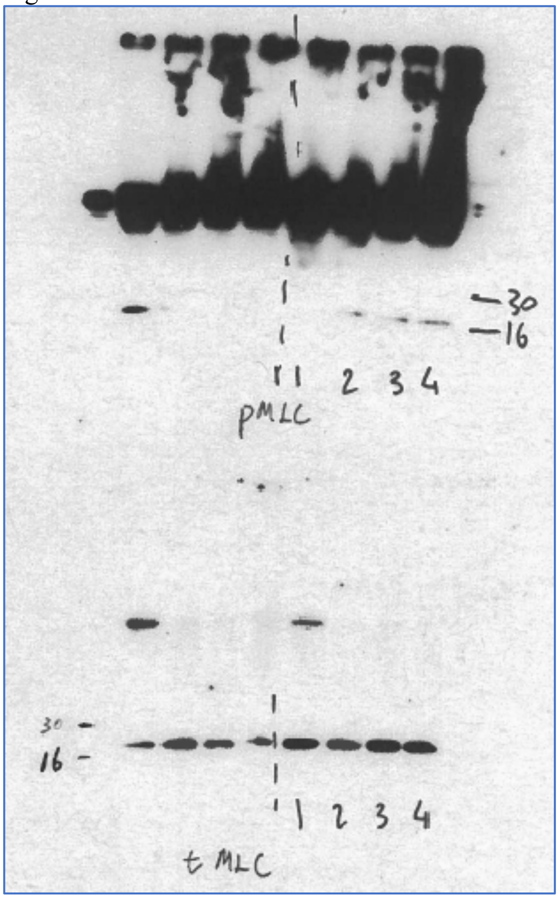

Fig. S8A

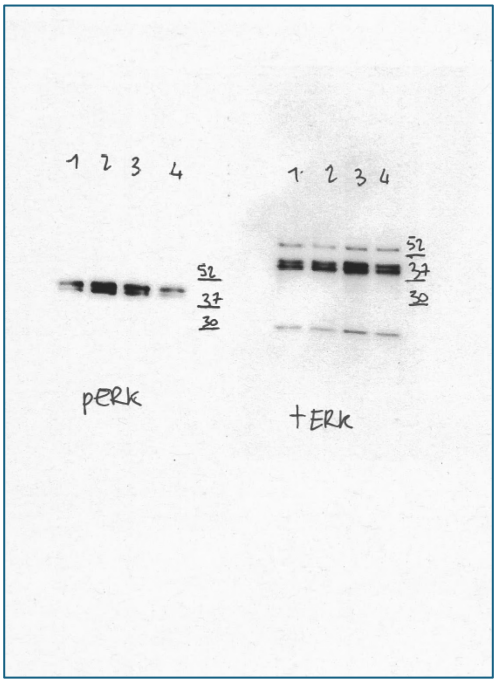

Fig. S9C

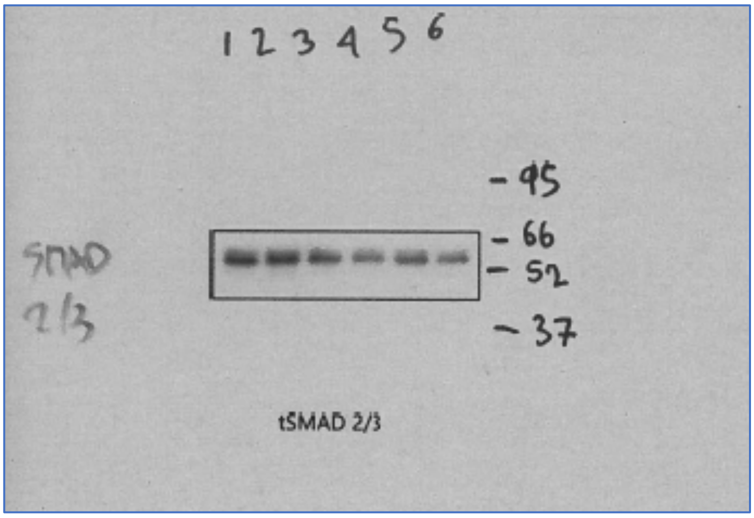

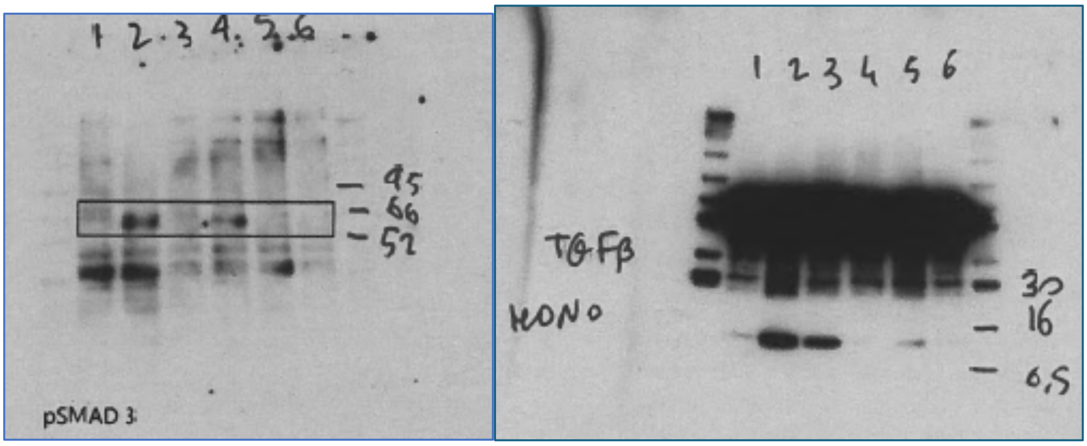

Fig. S10A

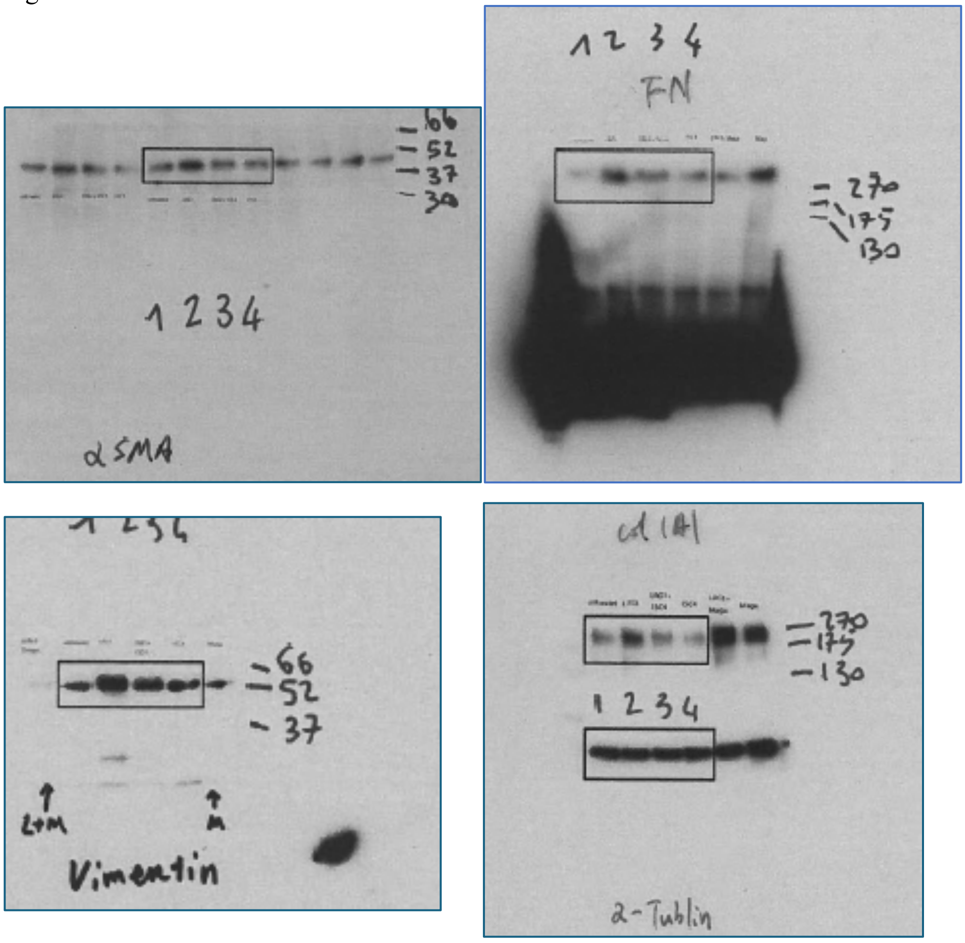

Data files

Data file S1. Raw data from experiments with n < 20
