# Supplementary material for: Leucine-rich α-2-glycoprotein 1 initiates the onset of diabetic retinopathy in mice
Source: Sci Transl Med. Author manuscript; Available in PMC 2025 Dec 8. (PMC7618449; doi:10.1126/scitranslmed.adn6047)
Supplement: Supplemental Material Reference [file EMS209684-supplement-Supplemental_Material_Reference.pdf]

67. S. Lorthois, F. Cassot, F. Lauwers, Simulation study of brain blood flow regulation by intra-cortical arterioles in an anatomically accurate large human vascular network: Part I: Methodology and baseline flow. *NeuroImage* **54**, 1031-1042 (2011).

68. R. Epp, F. Schmid, B. Weber, P. Jenny, Predicting Vessel Diameter Changes to Up-Regulate Biphasic Blood Flow During Activation in Realistic Microvascular Networks. *Frontiers in Physiology* **11**, (2020).

69. B. C. Fry, A. Harris, B. Siesky, J. Arciero, Blood flow regulation and oxygen transport in a heterogeneous model of the mouse retina. *Mathematical Biosciences* **329**, 108476 (2020).

70. P. W. Sweeney, S. Walker-Samuel, R. J. Shipley, Insights into cerebral haemodynamics and oxygenation utilising in vivo mural cell imaging and mathematical modelling. *Scientific Reports* **8**, 1373 (2018).

71. T. W. Secomb, R. Hsu, E. Y. H. Park, M. W. Dewhirst, Green's Function Methods for Analysis of Oxygen Delivery to Tissue by Microvascular Networks. *Annals of Biomedical Engineering* **32**, 1519-1529 (2004).

72. A. R. Pries, T. Secomb, T. Gessner, M. Sperandio, J. Gross, P. Gaehtgens, Resistance to blood flow in microvessels in vivo. *Circulation research* **75**, 904-915 (1994).

73. A. R. Pries, K. Ley, M. Claassen, P. Gaehtgens, Red cell distribution at microvascular bifurcations. *Microvascular research* **38**, 81-101 (1989).

74. A. S. Popel, Theory of oxygen transport to tissue. *Critical reviews in biomedical engineering* **17**, 257 (1989).

75. C. Magliaro, G. Mattei, F. Iacoangeli, A. Corti, V. Piemonte, A. Ahluwalia, Oxygen consumption characteristics in 3D constructs depend on cell density. *Frontiers in bioengineering and biotechnology* **7**, 251 (2019).

76. M. Berg, Y. Davit, M. Quintard, S. Lorthois, Modelling solute transport in the brain microcirculation: is it really well mixed inside the blood vessels? *Journal of Fluid Mechanics* **884**, A39 (2020).

77. A. R. Pries, T. W. Secomb, P. Gaehtgens, Biophysical aspects of blood flow in the microvasculature1. *Cardiovascular Research* **32**, 654-667 (1996).

78. Q. Fang, S. Sakadžić, L. Ruvinskaya, A. Devor, A. M. Dale, D. A. Boas, Oxygen advection and diffusion in a three-dimensional vascular anatomical network. *Opt. Express* **16**, 17530-17541 (2008).

79. A. Lücker, T. W. Secomb, B. Weber, P. Jenny, The relative influence of hematocrit and red blood cell velocity on oxygen transport from capillaries to tissue. *Microcirculation* **24**, e12337 (2017).

80. A. S. Golub, R. N. Pittman, Oxygen dependence of respiration in rat spinotrapezius muscle in situ. *American Journal of Physiology-Heart and Circulatory Physiology* **303**, H47-H56 (2012).

81. J. C. Cruz Hernández, O. Bracko, C. J. Kersbergen, V. Muse, M. Haft-Javaherian, M. Berg, L. Park, L. K. Vinarcsik, I. Ivasyk, D. A. Rivera, Y. Kang, M. Cortes-Canteli, M. Peyrounette, V.

Doyeux, A. Smith, J. Zhou, G. Otte, J. D. Beverly, E. Davenport, Y. Davit, C. P. Lin, S. Strickland, C. Iadecola, S. Lorthois, N. Nishimura, C. B. Schaffer, Neutrophil adhesion in brain capillaries reduces cortical blood flow and impairs memory function in Alzheimer's disease mouse models. *Nature Neuroscience* **22**, 413-420 (2019).

82. B. C. Fry, E. B. Coburn, S. Whiteman, A. Harris, B. Siesky, J. Arciero, Predicting retinal tissue oxygenation using an image-based theoretical model. *Mathematical Biosciences* **305**, 1-9 (2018).

83. A. L. Lyubarsky, L. L. Daniele, E. N. Pugh, From candelas to photoisomerizations in the mouse eye by rhodopsin bleaching in situ and the light-rearing dependence of the major components of the mouse ERG. *Vision Research* **44**, 3235-3251 (2004).

84. M. J. Albargothy, N. N. Azizah, S. L. Stewart, E. P. Troendle, D. H. W. Steel, T. M. Curtis, M. J. Taggart, Investigation of heterocellular features of the mouse retinal neurovascular unit by 3D electron microscopy. *Journal of Anatomy* **243**, 245-257 (2023).

85. K. Uchida, M. P. Reilly, T. Asakura, Molecular stability and function of mouse hemoglobins. *Zoological Science* **15**, 703-706 (1998).

86. A. Joseph, A. Guevara-Torres, J. Schallek, Imaging single-cell blood flow in the smallest to largest vessels in the living retina. *eLife* **8**, e45077 (2019).

87. I. Sencan, T. V. Esipova, M. A. Yaseen, B. Fu, D. A. Boas, S. A. Vinogradov, M. Shahidi, S. Sakadžic, Two-photon phosphorescence lifetime microscopy of retinal capillary plexus oxygenation in mice. *Journal of Biomedical Optics* **23**, 126501 (2018).
